# Predicting Clinical Sensitivities of *PDGFRA* Exon 18 Mutations to Imatinib and Avapritinib to Optimize Gastrointestinal Stromal Tumor Treatment

**DOI:** 10.1158/2767-9764.CRC-26-0093

**Published:** 2026-07-06

**Authors:** Homma M. Khosroyani, Alina Teuber, Ajia Town, Lillian R. Klug, Denisse Evans, Jerry Call, Sara Rothschild, Neeta Somaiah, Prapassorn Thirasastr, Mengyuan Liu, Ping Chi, Peter Hohenberger, Piotr Rutkowski, Patrick Schöffski, Abbas Agaimy, Mehdi Brahmi, Jonathan K. Killian, Garrett M. Frampton, Carol Beadling, Sebastian Bauer, Johanna Falkenhorst, Michael C. Heinrich

**Affiliations:** 1 Department of Hematology and Medical Oncology, Portland VA Health Care System and OHSU Knight Cancer Institute, Oregon Health and Science University, Portland, Oregon.; 2 Department of Medicine, Memorial Sloan Kettering Cancer Center, New York, New York.; 3 The Life Raft Group, Wayne, New Jersey.; 4 Department of Sarcoma Medical Oncology, The University of Texas MD Anderson Cancer Center, Houston, Texas.; 5 Division of Surgical Oncology, Medical Faculty Mannheim of University of Heidelberg, Mannheim, Germany.; 6 Department of Soft Tissue/Bone Sarcoma, Maria Sklodowska-Curie National Research Institute of Oncology, Warsaw, Poland.; 7 Department of General Medical Oncology, University Hospitals Leuven, Leuven, Belgium.; 8 Laboratory Experimental Oncology, Department of Oncology, KU Leuven, Leuven Cancer Institute, Leuven, Belgium.; 9 Institute of Pathology, Friedrich-Alexander-University Erlangen-Nürnberg (FAU), University Hospital Erlangen (UKER), Erlangen, Germany.; 10 Department of Medical Oncology, Cancer Research Centre of Lyon, Centre Léon Bérard, Lyon, France.; 11 Foundation Medicine Inc, Cambridge, Massachusetts.; 12 Knight Diagnostic Laboratories, OHSU Knight Cancer Institute, Portland, Oregon.; 13 Department of Medical Oncology and Sarcoma Center, West German Cancer Center, University Duisburg- Essen, Essen, Germany.

## Abstract

The most common platelet-derived growth factor receptor α (*PDGFRA*) alteration in gastrointestinal stromal tumors (GIST) is the exon 18 activation loop mutation D842V, which is resistant to imatinib and other type II tyrosine kinase inhibitors (TKI) but sensitive to type I TKI avapritinib. Avapritinib is FDA-approved for first-line treatment of all *PDGFRA* exon 18–mutant GIST cases but is only available for D842V-mutant cases outside the United States. Non-D842V exon 18–mutant GIST cases are understudied and lack evidence-based treatment guidelines. However, there are a few previous reports describing non-D842V exon 18–mutant patients with GIST who responded well to imatinib therapy. Given that imatinib is more tolerable, globally accessible, and significantly less expensive than avapritinib, we sought to define which patients could be treated with imatinib rather than avapritinib. We assembled a cohort of more than 1,000 *PDGFRA* exon 18–mutant GIST cases and identified that 78% of these mutations involved a key autoinhibitory aspartic acid residue at position 842. Using cell-based models, we demonstrated that imatinib sensitivity was dependent on the amino acid class of the 842-position residue, with all hydrophobic amino acids except alanine conferring resistance. In contrast, all 842-position mutations were avapritinib-sensitive. Structural modeling supported our biochemical results and revealed how 842-position mutations induce changes that can interfere with imatinib binding. Lastly, our biochemical data were validated using imatinib response data for first-line metastatic disease; patients with predicted exon 18–sensitive mutations had longer progression-free survival than patients with predicted imatinib-resistant mutations. These results provide key evidence that should be used to guide therapy selection for *PDGFRA*-mutant GIST.

**Significance:**

Biochemical and structural modeling of PDGFRA exon 18 842-position mutations allows for the prediction of clinical TKI responses. These data can be used to generate new treatment guidelines for *PDGFRA* exon 18–mutant GISTs and select optimal therapies for patients.

## Introduction

Gastrointestinal stromal tumors (GIST) are the most common type of soft tissue sarcoma and are thought to arise from the interstitial cells of Cajal located in the muscular layers of the gut (1, 2). As GISTs are untreatable with conventional chemotherapy or radiation, identifying the molecular drivers of this disease led to the development of targeted treatments that revolutionized the care for advanced and metastatic GISTs (3–7). The two most common molecular drivers are gain-of-function alterations in homologous type III receptor tyrosine kinases (RTK) KIT or platelet-derived growth factor receptor α (PDGFRA), accounting for 70% and 15% of all GISTs, respectively (8–12). These GISTs can be effectively treated with ATP-competitive tyrosine kinase inhibitors (TKI; refs. 11–13). Notably, imatinib, a type II TKI, has been extremely successful in treating exon 11 *KIT*-mutant GIST (14–20) and was FDA-approved in 2001 for first-line treatment of patients with advanced or unresectable GIST (21).

Most *KIT*-mutant GISTs harbor alterations in the exon 11 juxtamembrane domain (JMD), with only approximately 1% of cases having a primary mutation in the activation loop (exon 17; ref. 12). In contrast, most *PDGFRA*-mutant GISTs harbor genetic alterations that affect exon 18 kinase activation loop, with only a minority having exon 12 (JMD) or exon 14 (ATP-binding domain) alterations (12, 22, 23). The most common *PDGFRA* driver mutation found in GISTs is exon 18 D842V (9, 10, 24, 25). This mutation induces a structural change that causes the kinase to favor an active, DFG-in activation loop conformation (26–28). This structural change leads to imatinib and type II TKI resistance, as these TKIs can only bind to inactive, DFG-out kinase conformations (9, 29–32). For many years, patients with D842V-mutant GISTs had no effective treatment options, as all approved first-, second-, third-, and fourth-line therapies for GIST are all type II TKIs. This disparity led to the development of avapritinib, a type I TKI specifically designed to target PDGFRA D842V as well as the homologous *KIT* D816V mutation (33–35). Avapritinib was FDA-approved in 2020 for first-line treatment for all *PDGFRA* exon 18-mutant GISTs but is only approved to treat D842V-mutant GIST in some European countries and China and is entirely unavailable to all patients in many other countries (36–39).

Based on their relative rarity, *PDGFRA*-mutant GISTs are much less well studied than *KIT*-mutant GISTs, and among *PDGFRA*-mutant GISTs, the D842V mutation is the most extensively characterized. Other non-D842V exon 18 mutations have been reported, albeit these mutations are much less common (9, 10, 31). Exon 18 *PDGFRA*-mutant GISTs were previously thought to be universally imatinib-resistant, based on the notion that all activation loop mutations behave similarly to D842V, in stark contrast to exon 12 and exon 14 primary mutations, which are generally imatinib-sensitive (9, 30, 31). Although published clinical data of imatinib outcomes for non-D842V *PDGFRA*-mutant GIST are very limited, some provide evidence that a subset of these patients benefited from imatinib therapy. A 2012 study by Cassier and colleagues (31) reported that first-line imatinib efficacy in patients with *PDGFRA* D842V-mutant GIST was significantly less than that of patients with non-D842V tumors, reflected by a median progression-free survival (mPFS) of 2.8 months versus 28.5 months, respectively. Another study in 2016 by Yoo and colleagues (30) reported a similar result, in which patients with non-D842V *PDGFRA*-mutant GIST treated with imatinib experienced a longer mPFS than those with D842V-mutant GIST (29.5 months versus 3.8 months). There were only a few patients with non-D842V, exon 18–mutant GIST included in these studies, and as their mutation types were unique and diverse, it was difficult to extrapolate patterns in response. Although these studies provided clinical evidence that not all exon 18 mutations are imatinib-resistant, unfortunately, no clinical guidelines or extensive studies have defined which types of exon 18–mutant GIST would benefit from an alternative treatment like imatinib.

Therefore, we sought to identify the types of *PDGFRA* exon 18–mutant GISTs that could be treated with imatinib, which is more clinically available and has a superior long-term safety and tolerability profile compared with avapritinib (40–43). Although avapritinib has generally manageable toxicities, there is the risk of cognitive side effects not seen with imatinib and other GIST TKIs, such as memory impairment, encephalopathy, and central nervous system bleeding, which poses concerns for patients, their family members, and physicians alike (44). Determining whether imatinib could be used in lieu of avapritinib can provide patients with safety concerns or intolerance to avapritinib an alternative, efficacious treatment. Our study herein is the first comprehensive study of its kind, in which we developed *in vitro* models to characterize different types of *PDGFRA* non-D842V exon 18 mutations to predict which patients could benefit from imatinib and which require avapritinib. We assembled the largest *PDGFRA*-mutant GIST patient cohort to date and identified that most exon 18 mutations involve the 842-codon position, which is a key autoinhibitory residue in the PDGFRA activation loop (26, 28). We then developed Ba/F3 and Chinese hamster ovary (CHO) cell-based models to assess the sensitivities of various exon 18 mutations to imatinib and avapritinib. Using these models, we found that imatinib sensitivity/resistance was highly dependent on the amino acid occupying the 842-codon position, whereas avapritinib sensitivity was unaffected by these substitutions. We used *in silico* modeling to elucidate the mechanistic basis of how 842-position mutations affect imatinib binding and how this was correlated with our biochemical half maximal inhibitory concentration (IC_50_) results. Notably, cross-validating our *in vitro* predictions with real-world clinical data supported the clinical utility of these predictions and the translational relevance of our models. We demonstrate how understanding the biochemical consequences of mutations at the key autoinhibitory residue in the PDGFRA activation loop can be leveraged to provide effective yet more personalized and accessible treatments for patients with *PDGFRA*-mutant GIST.

## Materials and Methods

### Curation of a *PDGFRA*-mutant GIST database and frequency of mutations

Genetic profiling data, mainly from aggregated molecular pathology reports from patients with *PDGFRA*-mutant GIST, were curated from the American Association for Cancer Research (AACR) GENIE public dataset v18 (RRID: SCR_026217; ref. 45), Oregon Health and Science University, Foundation Medicine, and seven European Sarcoma Centers (Vall D-Hebron Institute of Oncology, University Hospital Erlangen, Sarcoma Center at the University Hospital Essen, KU Leuven, University Medicine Mannheim, University Hospital Münster, and Maria Sklodowska-Curie Memorial Cancer Center and Institute of Oncology). Ethical approval was granted by local review boards at each participating center, and written informed consent was waived. Local approval was in accordance with the Declaration of Helsinki and other local or international guidelines. Data were fully anonymized before our analysis, and all direct identifiers were removed. As the Oregon Health and Science University, Foundation Medicine, and the seven European Sarcoma Centers are not reporting institutions to the AACR GENIE project, we are confident that these cases are not duplicated within the GENIE database. Other public datasets, such as COSMIC, were not used to curate this database to prevent duplication of patient cases. Each primary mutation was classified into a group based on the exon in which it was found (e.g., V561D = exon 12, N659K = exon 14, and D842V = exon 18). To prevent double reporting of mutations, information from duplicate patient samples was excluded from the final cohort; however, multiple mutations reported from a single patient sample were annotated as being in *cis*. Cases with only silent single-nucleotide polymorphisms (SNP) were excluded from our analysis, as they do not affect the final PDGFRA protein sequence. To note, although mutations D842_H845del and I843_D846del are synonymous at the final amino acid protein level due to flanking Asp (D) residues at 842 and 846, we reported these mutations as two separate entities in our series based on the nucleotide change annotations.

### Cloning of *PDGFRA*-mutant lentiviral vectors

An adapted pLENTI-c-Myc-DDK-IRES-neo vector (OriGene #PS100081) with a full-length wild-type (WT) *PDGFRA* cDNA was used as a backbone to create the desired *PDGFRA* exon 18 842-position mutations with a NEBuilder HiFi DNA assembly/Gibson assembly protocol and synthesized oligos encoding the mutations of interest. Single-stranded oligos were designed with silent barcodes around the intended mutation at the 842 position and synthesized by either Integrated DNA Technologies or Eurofins. Both the *PDGFRA*-D842X and D842_D846delinsX mutant libraries were created using this NEBuilder method. Site-directed mutagenesis was used to create additional 842-position mutant vectors using the Agilent QuikChange Lightning Site-Directed Mutagenesis Kit (#210519) and standard protocol provided by Agilent. Oligos and sequences of each *PDGFRA* mutation created are listed in Supplementary Table S1.

Transformation of plasmids was done with high-efficiency NEB Stable Competent *Escherichia coli* (NEB, C3040) using the high-efficiency transformation protocol. After plating and colony growth overnight at 30°C on LB Agar plates with chloramphenicol (Teknova #L1997), colonies were selected for plasmid miniprep cultures in LB media + 34 μg/mL chloramphenicol to grow at 30°C in a shaking incubator for 12 to 16 hours, until confluency. Plasmid minipreps to extract DNA were done using the Zyppy Plasmid Miniprep Kit (Zymo Research #D4036), and all plasmids were Sanger sequenced to ensure proper vector construction and confirm each *PDGFRA* mutation of interest. This sequencing step was done for each PDGFRA mutation before creating lentivirus and cell lines.

### Primers used for PCR amplification and Sanger sequencing of *PDGFRA*

The sequences of the primers used to PCR amplify and sequence *PDGFRA* cDNA exons 1 to 23 are listed in Supplementary Table S2.

### Cell line authentication

Parental Ba/F3 (DSMZ #ACC-300, RRID: CVCL_0161) and CHO (ATCC #CCL-61, RRID: CVCL_0214) cell lines that were used to create human PDGFRA-mutant cell lines were tested to authenticate the species of origin. Authentication was performed through IDEXX BioAnalytics. Ba/F3 cell line species of origin was confirmed through CellCheck 19-mouse short tandem repeat profiling, and CHO cell line species of origin was confirmed through CO1 DNA barcoding and interspecies contamination tests. During this study, all cell lines derived from these parental cells were regularly tested for *Mycoplasma* contamination by PCR and using the SouthernBiotech *Mycoplasma* detection kit (#13100-01).

### Generation of lentivirus using HEK293Ta cells

Sequence-verified pLENTI *PDGFRA*-mutant plasmids were used to create replication-deficient lentivirus in HEK293Ta cells (GeneCopoeia #LT008, RRID: CVCL_BT05) using the Lipofectamine LTX (Invitrogen #15338100) and ViraPower (Invitrogen #K497500) lentiviral expression system manufacturing protocol. HEK293Ta cells were cultured in DMEM, 10% fetal bovine serum (FBS), and 1% penicillin/streptomycin at 37°C and 5% CO_2_ and plated 24 hours prior to transfection. Cells were grown to ∼60% confluency prior to transfection with plasmid DNA, Lipofectamine LTX (Invitrogen #15338100), ViraPower (Invitrogen #K497500), and OptiMem (Gibco #31985088). Twenty-four hours after transfection, fresh HEK293 media were put on cells. Twenty-four hours after media change, lentivirus was harvested and filtered through a 0.45-μm filter (Restek #26157) and used for immediate Ba/F3 or CHO cell transduction or frozen at −80°C for later use.

### Generation of *PDGFRA*-mutant driven, interleukin-3–independent Ba/F3 cell lines

Parental Ba/F3 cells were grown in RPMI-1640 medium with 10% FBS and 1% penicillin/streptomycin, supplemented with murine interleukin-3 (IL-3; 10 ng/mL; Cell Signaling Technology #8923C) at 37°C and 5% CO_2_. Spin inoculation for 1 hour at 3,000 RPM was used to transduce Ba/F3s with filtered lentivirus, polybrene (3 μg/mL), and murine IL-3 (10 ng/mL). After spin inoculation, cells were incubated at 37°C and 5% CO_2_. The following day, transduced cells were spun down and resuspended in fresh Ba/F3 media supplemented with murine IL-3 (10 ng/mL) and underwent antibiotic selection with 1.5 mg/mL of G418 (Sigma-Aldrich #G5013) for 7 to 9 days, until cells transduced with mock virus were completely depleted. Genomic DNA from IL-3–dependent Ba/F3s was sequence confirmed after selection to ensure no other mutations were introduced prior to the performance of limiting dilution assays.

To generate IL-3–independent cell lines that were dependent on PDGFRA-mutant kinase activity and expression for survival, sequence-confirmed G418-resistant Ba/F3 cells were plated in limiting dilutions (1,000-,100-, and 10-cells per well in 96-well plates) in media with the absence of IL-3 to trigger transformation. Plates were incubated at 37°C and 5% CO_2_ for 4 weeks and were checked once a week to identify cell growth. Wells with growth were moved to 12-well plates to allow the expansion of each clonal population before additional cell line characterization. Prior to TKI testing experiments, full sequencing of the *PDGFRA* cDNA was performed on each clonal, IL-3–independent Ba/F3 cell line to ensure no additional mutations were present. Cell lines were considered properly “transformed” if growth was supported without IL-3 and no other *PDGFRA* cDNA mutations were acquired during the limiting dilution assays.

### Generation of stably expressing *PDGFRA*-mutant CHO cell lines

Parental CHO cells were grown in Ham’s F12K medium with 10% FBS and 1% penicillin/streptomycin at 37°C and 5% CO_2_. CHOs were transduced with filtered lentivirus and polybrene (8 μg/mL) and grown at 37°C and 5% CO_2_ overnight. Next day, transduced cells were given fresh CHO media supplemented with 1 mg/mL of G418 (Sigma-Aldrich #G5013) for 7 to 9 days, until cells transduced with mock virus were completely depleted. Prior to subsequent experiments, full sequencing of the *PDGFRA* cDNA was performed for each cell line.

### Western blot analysis

For Ba/F3 IL-3–independent cell lines, cells were plated of at least 5 × 10^5^ cells per well to 12-well plates with standard Ba/F3 media (RPMI-1640, 10% FBS, 1% Pen/Strep) a day prior to drug treatment. The next day, wells were treated with various TKI doses (ranging from 0 to 2,000 nmol/L) for 90 minutes, using DMSO as a no-drug control (maximal DMSO concentration at any given point in the dosing period was 0.25%). For CHO cell lines, a day prior to drug dosing, cells were plated at a density of at least 3 × 10^5^ cells per well in a six-well plate with standard CHO media (F12K, 10% FBS, and 1% penicillin/streptomycin) with 500 μg/mL G418. The day of drug treatment, media were changed from all wells and were serum-starved with OptiMEM (Gibco #31985088) and treated with various TKI doses (ranging from 0 to 2,000 nmol/L) for 90 minutes, using DMSO as a no-drug control (maximal DMSO concentration at any given point in the dosing period was 0.25%).

After TKI treatment of 90 minutes, whole-cell protein lysates were harvested using HGNT lysis buffer and protease/phosphatase inhibitor (Cell Signaling Technology #5872). Protein concentrations were determined based on the Bradford dye-binding method using the Bio-Rad Protein Assay (Bio-Rad Laboratories #5000006). Identical amounts of proteins were loaded for each well and separated by SDS/PAGE, as described by Laemmli (46), using Bio-Rad 4-15% or 10% SDS-PAGE gels and then transferred to nitrocellulose membranes using the Bio-Rad TransBlot Turbo transfer system (Bio-Rad #1704271) and high molecular weight protocol. After transfer, nitrocellulose membranes were blocked in 5% BSA for 1 hour prior to primary antibody incubation. Primary antibodies used over the course of the study are as follows: phosphorylated tyrosine Y20 (BD Biosciences #610000, RRID: AB_397423), phosphorylated PDGFRA-Y849 (Cell Signaling Technology #3170, RRID: AB_2162348), phosphorylated STAT3-Y705 (Cell Signaling Technology #9131, RRID: AB_331586), phosphorylated AKT S473 (Cell Signaling Technology, RRID: AB_329825), phosphorylated MAPK1/2 Y202/204 (Cell Signaling Technology #9101, RRID: AB_331646), PDGFRA (Cell Signaling Technology #3174, RRID: AB_2162345), STAT3 (Cell Signaling Technology #4904, RRID: AB_331269), AKT (Cell Signaling Technology #9272, RRID: AB_329827), MAPK1/2 (Cell Signaling Technology #9102, RRID: AB_330744), and B-tubulin (Cell Signaling Technology #2146, RRID: AB_2210545). All primary antibodies were used at 1:1,000 dilutions in 5% BSA. Secondary mouse–horseradish peroxidase (HRP; Bio-Rad #1706516 RRID: AB_11125547) and rabbit-HRP (Bio-Rad #1706515 RRID: AB_11125142) conjugated antibodies, at 1:5,000 dilutions in 5% milk were used along with ECL detection reagent (Cytiva #GERPN3244) and SuperSignal West Pico PLUS Chemiluminescent Substrate (Thermo Scientific #34577) for visualization on the Bio-Rad ChemiDoc system (RRID: SCR_019690).

### Inhibitors used in this study

Imatinib (STI-571) was purchased from Selleck Chemicals (#S2475), and avapritinib (BLU-285) was purchased from MedChemExpress (#HY-101561). Both inhibitors were maintained in 10 mmol/L stocks in DMSO at −20°C.

### Calculating half maximal IC_50_ values from Western blots

Densitometry analysis in Bio-Rad Image Lab Software (RRID: SCR_014210) was used to determine the ratio of phosphorylated PDGFRA (pPDGFRA) to total PDGFRA expression and normalized to the no-drug lane data (e.g., 0 nmol/L = 100% phosphorylation). These ratios of pPDGFRA to total PDGFRA at each drug dose were then used to calculate the IC_50_ value of an inhibitor, along with 95% confidence intervals (CI) and standard errors of the mean (SEM), using nonlinear regression analysis in GraphPad Prism (RRID: SCR_002798).

### Cell viability assays

Cell viability assays were used to confirm the inhibitory effects of a given TKI dose on viability. These assays were performed with IL-3–independent Ba/F3 cell lines that were Sanger-sequenced to confirm the *PDGFRA* mutation prior to use. Cells were plated in biological triplicate wells with various doses (ranging from 0 to 1,000 nmol/L) of either imatinib or avapritinib. After 4 days (96 hours), cell viability was measured using the Guava EasyCyte (RRID: SCR_025377) and Guava ViaCount (Cytek #4000-0040) reagents, according to the manufacturer’s instructions. ViaCount reagent distinguishes viable and nonviable cells with a nuclear dye that only stains nucleated cells. Viability (100%) was normalized to control wells with no added drug (0 nmol/L). Relative viability % at each dose was calculated, and nonlinear regression analyses in GraphPad Prism (RRID: SCR_002798) were used to calculate the 50% growth inhibition concentration (GI_50_).

### Serum protein shift assay to define imatinib sensitivity versus resistance

#### For the Ba/F3 model

IL-3–independent Ba/F3 PDGFRA I843_D846del cells were grown to confluency, spun down, and the pellets were washed with sterile phosphate-buffered saline (PBS). Cells were resuspended in either standard Ba/F3 media with FBS (RPMI-1640, 10% FBS, and 1% penicillin/streptomycin) or Ba/F3 + human serum albumin–human ɑ-acid glycoprotein (HSA–AAG) media, which contained RPMI-1640, 341 μmol/L HSA (Sigma #A9511), 1 mg/mL AAG (Sigma #G9885), and 1% penicillin/streptomycin. After plating at least 300,000 cells per well with either FBS-containing or HSA–AAG-containing media, wells were treated with imatinib using six doses (ranging from 0 to 5,000 nmol/L) for 4 hours at 37°C. Each experiment was done in tandem for direct comparison of differences in drug IC_50_ value with serum proteins. After 4 hours, whole-cell protein lysates were harvested, Western blots were run, and membranes were probed for phosphorylated tyrosine (BD Biosciences #610000, RRID: AB_397423) and total PDGFRA (Cell Signaling Technology #3174, RRID: AB_2162345) and imaged using the same protocol described in the previous section.

Experiments of these direct comparisons were repeated at least three times to determine the calculated IC_50_ value for each experimental condition. The ratio of IC_50_ values measured in experiments using FBS-containing media versus HSA-AAG media gave the overall serum shift factor. This calculated shift factor was used to correlate IC_50_ value results from *in vitro* to make clinical predictions on whether tested variants can be considered as sensitive or resistant to a given TKI, a method adapted and further described in Liu and colleagues (47). To calculate the threshold IC_50_ value, we used the previously reported imatinib C_min_ (minimum blood plasma concentration reached by a drug during a dosing interval) in patients with GIST of 1,530 ng/mL from 400 mg once-daily imatinib dosing, which is the most common dose used for GIST treatment (47–49). This value was converted from ng/mL to nmol/L using imatinib’s molecular weight of 493.6 g/mol (1,530 ng/mL = 3,099.67 nmol/L) and then divided by the shift factor (3,099.67/16.4). This resulting 189 nmol/L concentration was used as the threshold IC_50_ value; values above this would be considered imatinib-“resistant,” and values below this value would be considered imatinib-“sensitive” (47).

#### For the CHO model

Cells were grown in standard CHO media (F12K, 10% FBS, and 1% penicillin/streptomycin) with 0.5 mg/mL G418 and plated in two- and six-well plates at a density of 500,000 cells per well a day prior to experiments. On the day of drug dosing, all 12 wells were washed with PBS to remove residual FBS serum. Next, either OptiMEM (Gibco) or OptiMEM + HSA–AAG media which contained OptiMEM, 341 μmol/L HSA (Sigma #A9511), and 1 mg/mL AAG (Sigma # G9885) was added to six wells each. Wells were then treated with imatinib using six doses (ranging from 0 to 5,000 nmol/L) for 90 minutes at 37°C. Each media condition set was done in tandem at the same time for direct comparison for differences in drug IC_50_ value with human serum proteins. Note here, an OptiMeM-only condition was used, as CHOs are incubated in this serum-free media during drug exposure to decrease the high background signal when imaging immunoblots. After 90 minutes, whole-cell protein lysates were harvested, Western blots were run, and membranes were probed for phosphorylated PDGFRA-Y849 (Cell Signaling Technology #3170, RRID: AB_2162348) and total PDGFRA (Cell Signaling Technology #3174, RRID: AB_2162345) and imaged using the same protocol described earlier. Experiments of these direct comparisons were repeated at least three times to determine the calculated IC_50_ value for each experimental condition. All calculations for the serum shift ratio and threshold value were done in the same manner as described earlier for the Ba/F3 model. For the CHO model, the shift factor was 1:40.4, and the calculated threshold value was 77 nmol/L.

### Survival curve analysis of the *PDGFRA*-mutant GIST cohort treated with first-line imatinib

Most of the genetic profiling data in the patient cohort were not associated with treatment response data, as these data were largely acquired from aggregated molecular pathology reports. The treatment data we used were sourced from patients who had metastatic GIST with a reported *PDGFRA* primary mutation and were treated with first-line imatinib. Data were fully anonymized before our analysis, removing all direct identifiers. Unpublished data of patient treatment responses were sourced from the following: The Life Raft Group GIST Registry (*n* = 19; ref. 50), different European sarcoma centers (*n* = 17), Oregon Health and Science University (*n* = 3), MD Anderson (*n* = 8), and Memorial Sloan Kettering Cancer Center (*n* = 2). We also incorporated data from previously published data from an European Organisation for Research and Treatment of Cancer (EORTC) study (*n* = 49; ref. 31), a published series from South Korea (*n* = 13; ref. 30), and the published B2222 phase II study of imatinib efficacy (*n* = 5; ref. 20) to increase the sample size. Patient genetic profiling data from the Life Raft Group, EORTC series, Korean series, B2222 study, MD Anderson, and Memorial Sloan Kettering were not included in the initial patient cohort to avoid double reporting in public databases such as AACR GENIE (RRID: SCR_026217).

Time to progression was determined as the time from the treatment start date (time 0) to the first radiographic/clinical progression or patient death. Mutations were grouped as follows: (i) D842V, (ii) predicted exon 18–sensitive, or (iii) predicted exon 18–resistant. Predicted exon 18–sensitive and –resistant mutation groups were based on the 842-position amino acid and its corresponding *in vitro* profiling data presented in the study. A combined resistant mutation treatment group was also generated and used for analysis by combining the D842V and predicted exon 18–resistant groups. Survival curve analysis between the different mutation groups was done using Kaplan–Meier analysis and log-rank (Mantel–Cox) testing in GraphPad Prism (RRID: SCR_002798) to calculate and assess statistical differences in PFS.

### *In silico* modeling and crystal structure data availability

PyMOL (W.L. DeLano, The PyMOL Molecular Graphics System, Schrödinger, RRID: SCR_000305) was used for generating the *in silico* models and figures. Previously reported crystal structures that were used for modeling and analysis are published (51) and deposited at the following RCSB Protein Data Bank (PDB; RRID: SCR_012820) ID accession codes: 8PQJ (https://doi.org/10.2210/pdb8pqj/pdb; ref. 51), 8PQK (https://doi.org/10.2210/pdb8pqk/pdb; ref. 51), 8PQH (https://doi.org/10.2210/pdb8pqh/pdb; ref. 51), and 6JOL (https://doi.org/10.2210/pdb6jol/pdb). For the visualization of the different PDGFRA mutants (i.e., D842D, D842V, D842I, D842A, and D842G), the mutagenesis tool of PyMOL (RRID: SCR_000305) was used, with all structures aligned to PDB-ID 8PQH (51).

### Statistical analysis and rigor

All statistical analysis was performed as indicated using GraphPad Prism 10.5 version software (RRID: SCR_002798) for both Windows and MAC OS X operating systems. The sample size for all experimental assays (e.g., immunoblotting, cell viability, and serum shift) was a minimum of three biological replicates.

## Results

### Mutations observed in the *PDGFRA* exon 18–mutant GIST cluster around the 842-codon position

**Figure 1.**
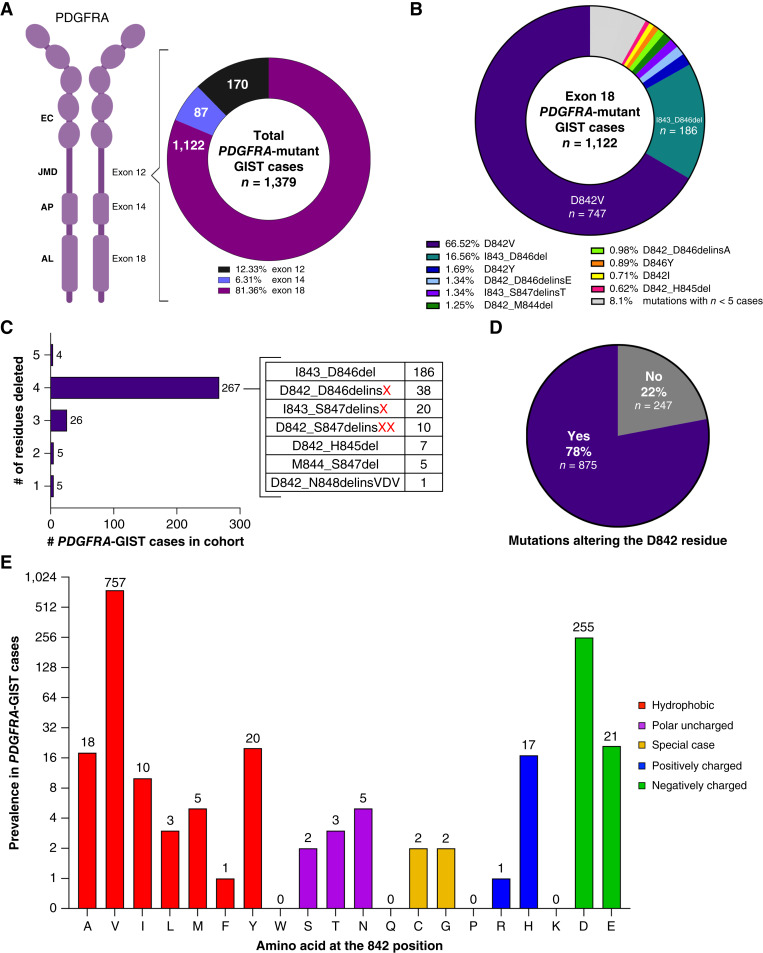
Mutation analysis of our *PDGFRA*-mutant GIST cohort reveals clustering around the exon 18, 842-codon position. **A,** The protein domains of PDGFRA (AL, activation loop; AP, ATP-binding domain; EC, extracellular domain) and the exon distribution of unique primary mutations seen in *PDGFRA*-mutant GIST (*n* = 1,379 cases). **B,** Breakdown of mutations seen in exon 18 *PDGFRA*-mutant GIST cases (*n* = 1,122/1,379). The gray-colored proportion in the chart indicates mutations with five or fewer reported cases (*n* = 90/1,122). **C,** Number of cases with exon 18 in/del mutations and the net number of deleted residues in the final protein sequence, along with the breakdown of the type of mutations observed with a 4-residue deletion. Red “X” denotes the number of amino acids inserted (X = 1, XX = 2). **D,** Proportion of cases with mutations that directly alter the 842 residue. **E,** Distribution of the amino acid occupying the 842-position in exon 18–mutant cases (*n* = 1,122). Colors in the legend correspond to the amino acid class of the 842-position residue (hydrophobic, polar uncharged, special case, positively charged, and negatively charged). [Portions of **A** were Created in BioRender. Khosroyani, H. (2026) https://BioRender.com/l88qy7p.]

First, we determined the frequency and spectrum of primary *PDGFRA* mutations observed in GIST by compiling a deidentified database using genetic profiling information from Oregon Health and Science University, the AACR GENIE project v18 (45), Foundation Medicine, and a European consortium of academic sarcoma centers (Supplementary Table S3). Our final cohort included 1379 *PDGFRA*-mutant GIST cases. The majority of cases harbored primary genomic alterations in the activation loop (exon 18, 81.36%, *n* = 1,122/1,379), with the remaining having alterations in the juxtamembrane domain (exon 12, 12.33%, *n* = 170/1,379) or the ATP-binding domain (exon 14, 6.31%, *n* = 87/1,379; Fig. 1A; Supplementary Table S3). Genomic alterations such as silent SNPs that did not alter the final protein sequence were excluded. Our final cohort of 1,122 cases of exon 18 *PDGFRA*-mutant GIST were further analyzed. A total of 747 cases in this cohort (66.58%, *n* = 747/1,122) had D842V, whereas 193 cases harbored the second most common *PDGFRA* mutation, I843_D846 deletion (17.27%, *n* = 193/1,122; Fig. 1B). In addition, there were a variety of non-D842V point mutations and complex insertion/deletion (in/del) mutations observed (Fig. 1C). Among these, 307 cases had in/del mutations with at least one deleted residue, with 267 of them having a net deletion of four residues (Fig. 1C). Although approximately 70% of these cases had I843_D846del (*n* = 186/267), we still observed numerous other in/dels around D842 that resulted in a net deletion of four residues from the final protein sequence (Fig. 1C). Overall, approximately 78% (*n* = 875/1,122) of the mutations found in our exon 18 patient series directly altered the 842 residue (Fig. 1D), which is an important autoinhibitory residue involved in stabilizing an inactive kinase conformation (28, 52, 53).

The binding of ATP-competitive, type II TKIs like imatinib are highly dependent on kinase structure; therefore, overall structural changes can drastically alter whether they effectively inhibit aberrant kinase activity (54–56). D842V-mutant GISTs are resistant to type II TKIs; however, *PDGFRA*-mutant GISTs with the second most common mutation, exon 18 I843_D846del, respond to imatinib treatment (9, 30). A key difference between these two mutations when comparing the final protein sequence, besides the 4-residue deletion, is the resultant amino acid in the 842 position. The change from negatively charged Asp (D) to a hydrophobic Val (V) in D842V differs from I843_D846del, whereas the amino acid at the 842 position remains the WT Asp (D). Whereas V and D were the most commonly observed amino acids at the 842 position in our patient cases, all other amino acid substitutions were observed at the 842 position at least once in our series except for W, Q, P, and K (Fig. 1E). As there are significant changes to the 842-position amino acid that correspond to polarizing changes in clinical imatinib responses between D842V-mutant tumors and I843_D846del-mutant tumors, we hypothesized that this 842-position amino acid played a key role in determining imatinib sensitivity and that sensitivity/resistance was independent of deleted adjacent residues. We further hypothesized that amino acids within each class (hydrophobic, positively charged, negatively charged, polar uncharged, and special case) would share class-specific relative imatinib sensitivities when located in the 842 position. As nearly every single amino acid had been observed at the 842 position in our patient series, we believe that modeling every possible 842-position mutation would allow us to predict imatinib sensitivities of the non-D842V cases in our cohort.

### Validating the effects of GIST TKIs on clinically observed exon 18 *PDGFRA* mutations

Before directly testing our hypothesis, we validated our *in vitro* systems using clinically imatinib-resistant *PDGFRA* D842V and imatinib-sensitive *PDGFRA* I843_D846del mutations as controls. As there are no available *PDGFRA*-mutant GIST immortalized cell lines, we chose to use Ba/F3 and CHO cell-based models to study our mutations of interest, as both are widely used in GIST research. First, we modeled mutations in Ba/F3 cells, a murine pro-B cell line that is a popular model system for studying oncogenic kinases (57). Parental Ba/F3s are dependent on IL-3 for survival but can become “transformed” by human oncogenes and become dependent on the activity of a transgene to confer IL-3–independent growth (57–59). We used this model system to test the inhibitory and growth effects of TKIs on different *PDGFRA* mutations. Ba/F3 cells were transduced with lentivirus encoding human *PDGFRA* cDNA with D842V or I843_D846del mutations and subsequently subjected to IL-3 withdrawal transformation assays, as further described in the “Materials and Methods”. Isolated clonal cell lines that proliferated without IL-3 and with only the intended *PDGFRA* mutation were considered “transformed” and selected for further TKI testing. Clonal cell lines with additional *PDGFRA* mutations were not considered as transformed, as additional coding sequence mutations could influence protein structure and TKI binding in unpredictable ways.

**Figure 2.**
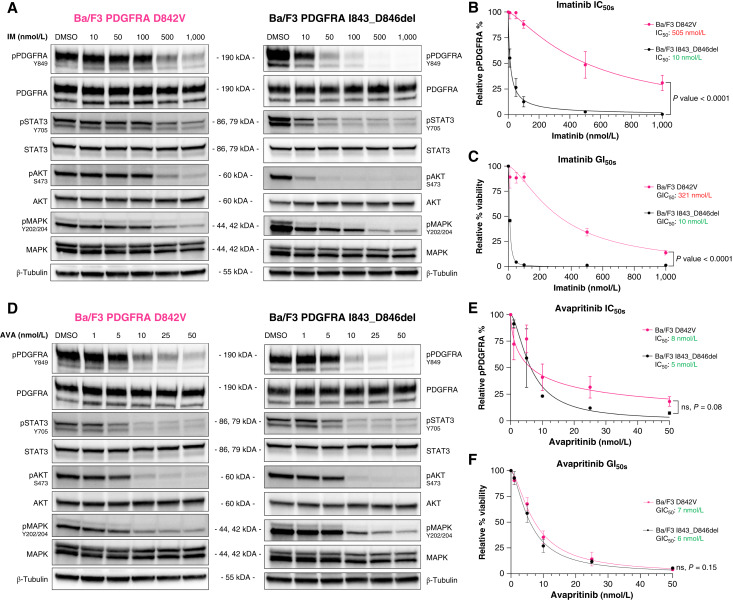
Modeling *PDGFRA* D842V and *PDGFRA* I843_D846del mutations in Ba/F3 cells recapitulate clinical imatinib and avapritinib sensitivity/resistance. Immunoblot analysis and quantification of IL-3–independent Ba/F3 D842V and I843_D846del cell lines. Equal amounts of lysates harvested after 90 minutes of exposure to varying doses of imatinib (IM) and avapritinib (AVA) were loaded for each dose. Data represent the results of at least three independent experiments. **A,** Representative immunoblots of Ba/F3 D842V and I843_D846del cells treated with IM are shown. Immunoblotting for pPDGFRA, phosphorylated STAT3 (pSTAT3), phosphorylated AKT (pAKT), and phosphorylated MAPK1/2 (pMAPK1/2) and corresponding total protein expression is shown along with a β-tubulin loading control. **B,** Relative % pPDGFRA is graphed after treatment of Ba/F3 D842V and I843_D846del cells with IM, quantified as the ratio of pPDGFRA to total PDGFRA at each drug dose from at least three independent immunoblotting experiments. Imatinib IC_50_ values were calculated using nonlinear regression analysis, with the *P* value corresponding to an extra sum of squares F test, testing whether the difference between the IC_50_ values was statistically significant. **C,** Relative % viability of Ba/F3 D842V and I843_D846del cells after 96 hours of treatment with IM; each point is the mean calculated viability ± SEM from at least three independent experiments. The GI_50_ value was calculated using nonlinear regression analysis. The *P* value from an extra sum of squares F test was used to determine whether the difference in GI_50_ values was statistically significant. **D,** Representative immunoblots of Ba/F3 D842V and I843_D846del cells treated with AVA. Blots with pPDGFRA, pSTAT3, pAKT, and pMAPK1/2 and corresponding total protein expression, along with β-tubulin, are shown. **E,** Relative % pPDGFRA is graphed after AVA treatment of Ba/F3 D842V and I843_D846del cells. Quantification, analyses, and IC_50_ value calculations are the same as in **B**. *P* value (0.08) from an extra sum of squares F test indicates that the difference in IC_50_ values between the two cell lines was not significant. **F,** Relative % viability of Ba/F3 D842V and I843_D846del cells after 96 hours of AVA treatment; each point is the calculated viability ± SEM from at least three independent experiments. The GI_50_ value was calculated using nonlinear regression analysis in GraphPad Prism. The *P* value (0.15) from an extra sum of squares F test indicates that the difference in GI_50_ values between the two cell lines was not significant.

To validate whether the IL-3–independent Ba/F3 D842V and I843_D846del cell lines recapitulate clinical drug responses, immunoblotting was performed with various doses of imatinib and probed for activated pPDGFRA and total PDGFRA, along with downstream signaling molecules. Drug IC_50_ values were calculated based on the degree of pPDGFRA inhibition relative to total PDGFRA expression. As expected, D842V cells required higher imatinib concentrations to inhibit pPDGFRA and activated downstream effectors compared with I843_D846del cells (Fig. 2A; Supplementary Fig. S1A and S1B). Imatinib IC_50_ values for D842V and I843_D846del were 505 and 10 nmol/L, respectively (Fig. 2B). Next, assays were performed to determine whether drug-induced inhibition of pPDGFRA and subsequent downstream signaling resulted in decreased viability. Viability measurements after 96 hours of imatinib treatment revealed that 50% of growth was inhibited (GI_50_) at 321 nmol/L for D842V cells, compared with 10 nmol/L for I843_D846del cells, mirroring IC_50_ trends (Fig. 2C). Avapritinib was also tested to further validate our model, as both D842V and I843_D846del are sensitive to this drug. Ba/F3 D842V and I843_D846del cells both displayed significant inhibition of pPDGFRA and activated downstream signaling at low avapritinib concentrations (Fig. 2D; Supplementary Fig. S1C and S1D), with IC_50_ values for D842V and I843_D846del of 8 and 5 nmol/L, respectively (Fig. 2E). The potent inhibition of pPDGFRA with avapritinib was also reflected in a decrease of cell viability in both D842V cells and I843_D846del cells at low concentrations, with GI_50_ values of 7 and 6 nmol/L, respectively (Fig. 2F).

We also used CHO cells to model *PDGFRA* mutations that we were not transforming in our BaF3 model system. Unlike Ba/F3s, the creation of stably expressing human *PDGFRA* mutations in CHOs is not dependent on mutant activity for survival, but TKI potency testing is still possible as CHOs robustly express exogenous proteins of interest. We created stably expressing PDGFRA D842V and PDGFRA I843_D846del CHO cells and validated the inhibitory effects of imatinib and avapritinib on pPDGFRA using immunoblotting. Imatinib IC_50_ values recapitulated clinically resistant and sensitive responses at 582 and 12 nmol/L for D842V and I843_D846del, respectively (Supplementary Fig. S2A and S2B). Avapritinib IC_50_ values also recapitulated clinically sensitive responses at 5 nmol/L for D842V and 4 nmol/L for I843_D846del (Supplementary Fig. S2C and S2D). With these complementary models, we validated the use of immunoblotting with the measurement of pPDGFRA inhibition as a surrogate for determining cellular sensitivity and resistance to TKIs.

As our overall goal was to classify *PDGFRA* 842-position mutations as imatinib-“sensitive” or imatinib-“resistant” to predict clinical response, we needed to define a threshold IC_50_ value of clinical resistance in our *in vitro* systems. *In vitro* growth conditions are different than human physiologic conditions; therefore, we next determined whether the IC_50_ calculations were different between *in vitro* serum media conditions (FBS in Ba/F3s, OptiMEM in CHOs) or human physiologic serum conditions, as TKIs can bind with different affinities to human serum proteins versus serum used in standard culture media (47). We used physiologic concentrations of HSA and AAG for our experiments, as both proteins are important in drug binding, transporting, and distributing various classes of drugs, including TKIs (60). We tested these conditions in our validated imatinib-sensitive Ba/F3 I843_D846del cells and CHO I843_D846del cells. By performing parallel immunoblotting experiments in standard cell media or media with HSA–AAG, we directly evaluated the changes in imatinib IC_50_ values. There was a significant difference in the inhibition of pPDGFRA and imatinib IC_50_ values between *in vitro* and human physiologic conditions in both Ba/F3 and CHO I843_D846del cells, with the calculated imatinib serum shift factor at a 1:16.4 ratio in the Ba/F3 model (Supplementary Fig. S3A and S3B) and 1:40.4 in the CHO model (Supplementary Fig. S3C and S3D). As further described in the methods, these ratios were used along with a previously reported GIST imatinib C_min_ (minimum drug concentration in plasma between doses) of 1,530 ng/mL to calculate a threshold IC_50_ value for clinical resistance (47, 49). In the Ba/F3 model, the threshold was 189 nmol/L, whereas in the CHO model, the threshold was 77 nmol/L. We classified mutations as resistant or sensitive based on where their IC_50_ values ± 95% CIs fell in relation to these threshold IC_50_ values.

### Imatinib and avapritinib sensitivities of all possible amino acid substitutions at the *PDGFRA* exon 18 842 position

**Figure 3.**
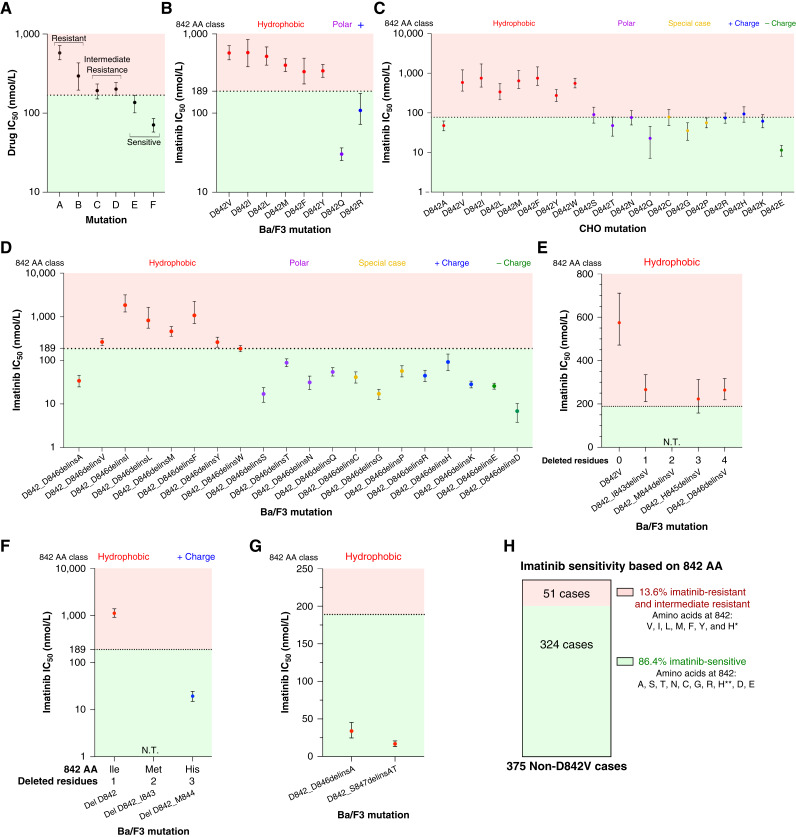
Profiling imatinib sensitivities of stably expressing IL-3–independent Ba/F3 and CHO *PDGFRA* 842-mutant cell lines. Immunoblot quantification of the effect of imatinib on pPDGFRA in Ba/F3 and CHO cells. The ratio of pPDGFRA to total PDGFRA across doses was calculated and used with nonlinear regression analyses in GraphPad Prism to determine IC_50_ values and 95% CIs. The horizontal dotted line in **B** and **D–G** is at 189 nmol/L, a calculated threshold IC_50_ value separating predicted clinical resistance vs. clinical sensitivity in the Ba/F3 model, and in **C**, the threshold IC_50_ value is at 77 nmol/L for the CHO model. **A,** Key for interpreting and classifying mutant IC_50_ values, with error bars indicating the 95% CI of the IC_50 value_. Resistant mutations will have IC_50_ values and 95% CIs only within the shaded red area, whereas sensitive mutations will have IC_50_ values and 95% CIs only within the shaded green area. Intermediately resistant mutations will have IC_50_ values and/or 95% CIs within both the green and red shaded areas. Imatinib IC_50_ values are shown for (**B**) Ba/F3 cells expressing PDGFRA D842X mutations, (**C**) CHO cells expressing PDGFRA D842X mutations, (**D**) Ba/F3s expressing all possible, 4-residue deletion D842_D846delinsX mutations, and (**E**) Ba/F3s expressing mutations with 0–4 deleted residues and Val in the 842-position, with “N.T.” denoting a nontransforming mutation. **F,** Ba/F3s expressing mutations with 1-, 2-, or 3-residue deletions at the 842 position, where the resulting 842 amino acid is the next residue in the WT PDGFRA sequence, with “N.T.” denoting a nontransforming mutation. **G,** Ba/F3s expressing 842-position Ala mutations in a 4-residue deletion context. In **B–G**, the error bars represent the 95% CI, with colors of dots indicating the amino acid (AA) class that is in the 842-position (red, hydrophobic; purple, polar uncharged; yellow, special case; blue, positively charged; and green = negatively charged). **H,** Proportion of 375 non-D842V cases from the 1,122 patient cohort that would have predicted imatinib-sensitive or -resistant/intermediate resistant mutations based on the 842-position amino acid IC_50_ value results from **B–G**. *indicates D842H mutation, and **indicates in/del mutations with H in the 842-position.

After validating the effects of TKIs and determining the threshold of sensitivity versus resistance for each cell-based model, we tested our hypothesis that the 842-position amino acid determines imatinib sensitivity by first creating libraries of human *PDGFRA*-mutant cDNA in a pLENTI-neo backbone vector with every possible amino acid, including stop codons, at the 842 position, in both a point mutation and a 4-residue deletion context (D842_D846delinsX). The 4-residue deletion backbone with I843-D846 deleted residues was used, as this was the most common series of residues deleted in the mutations observed in our cohort (Fig. 1C). These lentiviral vector libraries were used to create individual cell lines, as described in the methods section. In the D842X point mutation context, only eight (V, I, L, M, F, Y, Q, and R) amino acid substitutions successfully transformed Ba/F3 cells and were driven by mutant PDGFRA activity. As expected, the nononcogenic variants WT (D842D) and D842* (stop) did not cause transformation. The 11 other nontransforming D842X mutations were instead modeled in CHOs. In contrast, in the D842_D846delinsX 4-residue deletion context, all 20 amino acid substitutions, but not including the stop codon, transformed Ba/F3s and were only tested in this model. Mutant IC_50 *values*_ were calculated using immunoblotting after treating Ba/F3 and stable CHO cell lines with imatinib. Mutations were classified as sensitive, intermediately resistant, or resistant. Resistant mutations were defined as having IC_50_ values and 95% CIs fully above the threshold value, whereas sensitive mutations were defined as having IC_50_ values and 95% CIs fully below the threshold value (Fig. 3A). Mutations with IC_50_ values whose 95% CIs crossed the threshold value were classified as having intermediate resistance (Fig. 3A).

D842X mutations with hydrophobic amino acids V, I, L, M, F, Y, or W were imatinib resistant, with calculated IC_50_ values, 95% CIs, and SEMs that were fully above the threshold value (189 nmol/L for the Ba/F3 model, 77 nmol/L for CHOs; Fig. 3B and C; Supplementary Fig. S4A and S4B). However, the hydrophobic D842A mutation was imatinib-sensitive, thus differing from all other hydrophobic D842X mutations (Fig. 3B; Supplementary Fig. S4B). Imatinib sensitivities for other D842X mutations were mostly similar across other amino acid classes and differed from the more resistant hydrophobic residue mutations. Within the polar uncharged group, Q was sensitive, but S, T, and N exhibited intermediate resistance (Fig. 3B and C; Supplementary Fig. S4A and S4B). For special case amino acids, G and P were classified as sensitive, whereas C displayed intermediate resistance (Fig. 3C; Supplementary Fig. S4B). Positively charged R was classified as sensitive in Ba/F3s but displayed intermediate resistance in CHOs (Fig. 3B and C; Supplementary Fig. S4A and S4B). D842X substitution mutations with positively charged H and K exhibited intermediate resistance (Fig. 3B and C; Supplementary Fig. S4B). Lastly, the D842X mutation to negatively charged E, the same amino acid class as WT D, was imatinib-sensitive (Fig. 3C; Supplementary Fig. S4B).

Imatinib IC_50_ values between D842X mutations to their 4-residue deletion mutation counterparts followed similar trends. D842_D846delinsX-mutant kinases with the 842 position occupied by amino acids in the polar uncharged (S, T, N, and Q), special case (C, G, and P), positively charged (R, H, and K), and negatively charged (D and E) classes were imatinib-sensitive (Fig. 3D; Supplementary Fig. S4C). Hydrophobic amino acid substitutions to V, I, L, M, F, and Y conferred imatinib resistance, whereas the W substitution exhibited intermediate imatinib resistance (Fig. 3D; Supplementary Fig. S4C). Strikingly, as seen with D842A, the D842_D846delinsA mutation conferred imatinib sensitivity, displaying a sensitivity profile different from the other hydrophobic 842-substitution mutations (Fig. 3C and D; Supplementary Fig. S4B and S4C).

Next, we tested additional mutations to determine whether the number of deleted residues adjacent to the 842 position significantly altered imatinib sensitivity. As D842V and D842_D846delinsV were profiled above, we chose to model 1-, 2-, or 3-residue deletions with the 842 position mutated to V (e.g., D842_I843delinsV, D842_M844delinsV, D842_H845delinsV, and D842_D846delinsV) in Ba/F3s. The D842_M844delinsV mutation was not transforming and was not further modeled, as this mutation has never been clinically observed. We discovered that regardless of the number of adjacent deleted residues, when V occupies the 842 position, the resulting mutant kinase was either imatinib-resistant (e.g., D842V, D842_I843delinsV, and D842_D846delinsV) or displayed intermediate resistance (e.g., D842_H845delinsV; Fig. 3E; Supplementary Fig. S4D).

To further test our hypothesis of the importance of the amino acid at the 842 position, we used Ba/F3s to profile the imatinib sensitivity of mutant kinases with 1-, 2-, or 3-residue deletions starting at the 842 position, with the resultant amino acid in the 842 position being the next residue in the WT sequence. The resulting amino acids at the 842 position were I (D842del), M (D842_I843del), and H (D842_M844del). The D842_I843del mutation was nontransforming in Ba/F3s and was not further tested. The D842del mutation conferred imatinib resistance, whereas the D842_M844del mutation conferred imatinib sensitivity (Fig. 3F; Supplementary Fig. S4E), concordant with previous results for other in/del mutations with hydrophobic I and positively charged H in the 842 position, respectively (Fig. 3B–D; Supplementary Fig. S4A–S4C).

In addition, as Ala (A) was the only hydrophobic amino acid at the 842 position to confer imatinib sensitivity, we profiled an additional clinically observed mutation, D842_S847delinsAT, to determine whether this was also imatinib-sensitive. Notably, D842_S847delinsAT also conferred imatinib sensitivity (Fig. 3G; Supplementary Fig. S4F), further demonstrating that Ala (A) at the 842 position has different effects on a mutant PDGFRA kinase than other hydrophobic amino acid mutations. All imatinib IC_50_ values and corresponding 95% CIs for all mutations tested are listed in Supplementary Table S4. These data support our hypothesis that regardless of deleted residues in the final protein sequence, the 842-position amino acid plays a critical role in determining imatinib sensitivity and that amino acids in the same classes elicit similar sensitivities (apart from Ala).

We next confirmed the potency of avapritinib against these *PDGFRA* exon 18 mutations in both Ba/F3 and CHO systems. Avapritinib overcomes type II TKI resistance in D842V-mutant tumors by binding to a DFG-in, active conformation caused by the mutation (33, 34). Due to this binding characteristic, theoretically, avapritinib could be used to treat all PDGFRA exon 18–mutant kinases, although this theory has never been formally tested. We used 26 nmol/L as the IC_50_ threshold value for avapritinib resistance in the Ba/F3 model, an IC_50_ value we previously published for a Ba/F3 PDGFRA D842V + V658A cell line (37). This compound mutation was observed in patients with avapritinib-resistant tumors and currently has the lowest reported IC_50_ value of any avapritinib-resistant kinase (37). The threshold value was 80 nmol/L in the CHO model, set by the IC_50_ value for a PDGFRA D842V + V658A CHO cell line (Supplementary Fig. S5A). Regardless of the 842-position amino acid and imatinib sensitivity or resistance, all tested mutations were avapritinib sensitive, with no 95% CIs or SEMs crossing above the 26 nmol/L threshold in the Ba/F3 model, or 80 nmol/L in the CHO model (Supplementary Figs. S5B, S5C, and S6A–S6F). There was no difference in avapritinib sensitivity for D842X point mutations, their 4-residue deletion counterparts, and other 842-position mutations with additional deletion sizes (Supplementary Figs. S5B, S5C, and S6A–S6F). Avapritinib IC_50_ values and corresponding 95% CIs for all mutations tested are listed in Supplementary Table S5. These results provide the first biochemical data to support the theory that avapritinib could be used to treat any primary *PDGFRA* exon 18–mutant GIST, especially non-D842V, imatinib-resistant mutations.

Unfortunately, many patients outside of the United States do not have access to avapritinib due to country-specific regulatory actions; therefore, we used our cell-based IC_50_ data to predict how many non-D842V, exon 18 cases in our cohort could benefit from first-line imatinib therapy. A total of 375 cases in our cohort were reported to have non-D842V mutations. Of these, we predicted that 86.4% (*n* = 324/375) had mutations that would be responsive to imatinib (Fig. 3H; Supplementary Table S6). These cases had mutations with A, S, T, N, C, G, R, D, or E at the 842 position (Fig. 3H; Supplementary Table S6). Cases with in/del mutations with H in the 842 position were classified as imatinib-sensitive and included in this group. The remaining 13.6% of non-D842V cases were predicted to be imatinib-resistant and most likely unresponsive to this drug *in vivo*, as they had mutations with hydrophobic V, I, L, M, F, or Y residues in the 842 position (Fig. 3H; Supplementary Table S6). As D842H was classified as intermediately resistant based on our *in vitro* results, cases with this mutation were included in the predicted imatinib-resistant group (Fig. 3H; Supplementary Table S6).

### *In silico* modeling reveals that *PDGFRA* D842X point mutations affect activation loop flexibility

Although our *in vitro* work largely supported our hypothesis that 842-position amino acid substitutions within the same class elicit similar imatinib sensitivities, 842-position Ala (A) mutations were an exception. 842-Position Ala (A) mutations were observed in 18 cases in our cohort and was the only hydrophobic amino acid that did not confer imatinib resistance when located in the 842 position in either a point mutation (CHO: D842A) or in/del mutation (Ba/F3: D842_D846delinsA and D842_S847delinsAT) context. Therefore, we investigated whether structural changes could explain why 842-A mutations confer imatinib sensitivity compared with other hydrophobic mutations like D842V. Prior modeling studies have shown that imatinib lacks inhibitory activity against D842V because this mutation shifts the equilibrium to favor a DFG-in, active kinase conformation, inducing a structure that clashes with imatinib binding (28, 61). In contrast, imatinib is potent against the I843_D846del mutant kinase; therefore, it was theorized that I843_D846del favors an overall inactive conformation (61), while still allowing sufficient adoption of the active conformation in the absence of ligand binding to be oncogenic in the precursor cells that give rise to GIST. We hypothesized that 842-position Ala (A) mutations must not induce a structure that shifts the kinase state equilibrium toward a predominantly active state like D842V, and in turn, adopt a structure that allows for imatinib binding. To explore our hypothesis, we used *in silico* modeling based on publicly available crystal structures (51) to model the kinase domains of D842A and D842V to determine whether the changes seen in biochemical imatinib IC_50_ values might be explained by structural analysis. In addition, imatinib-sensitive D842G was modeled as G is even smaller than A and lacks a methyl sidechain, and imatinib-resistant D842I was modeled as I is also a hydrophobic amino acid but has one more methyl group than V.

**Figure 4.**
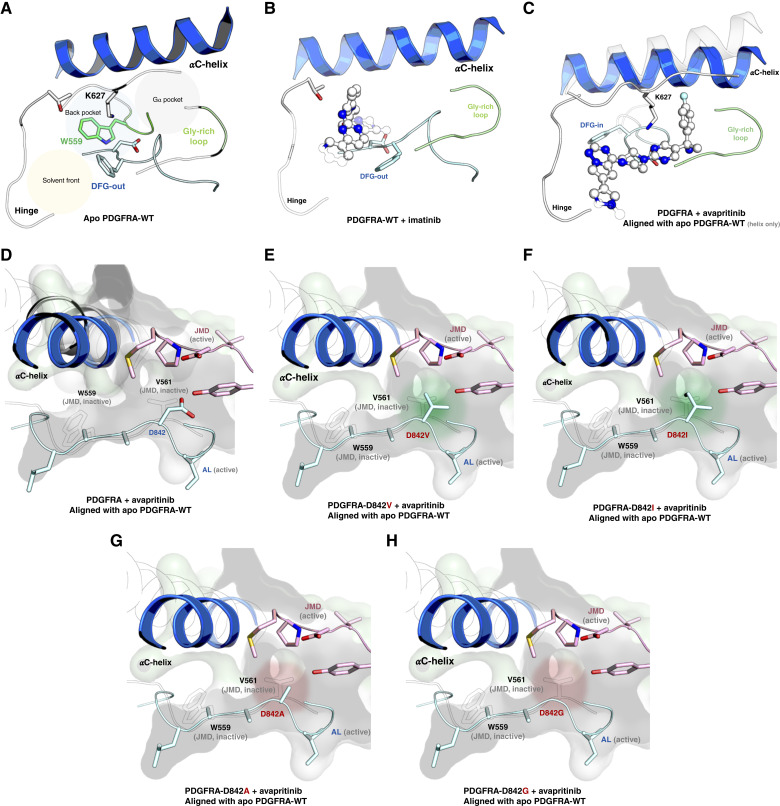
Binding modes of imatinib and avapritinib to PDGFRA-WT, along with activation loop visualization of PDGFRA D842X mutations modeled *in silico*. All visualizations shown were done in PyMOL, and all structures shown are aligned with apo PDGFRA-WT (PDB: 8PQJ). **A,** Visualization of apo PDGFRA-WT (PDB: 8PQJ), with the DFG motif in the out conformation and W559 protruding into the back pocket of the protein kinase domain. **B,** Visualization of the DFG-out motif within the PDGFRA activation loop and imatinib occupying the hydrophobic back pocket of PDGFRA-WT (WT; PDB: 6JOL). **C,** Visualization of the DFG-in motif within the PDGFRA activation loop bound to avapritinib (PDB: 8PQH), showing how avapritinib occupies the solvent front and Gα pocket but not the hydrophobic back pocket. *In silico* modeling based on the PDGFRA-T674I crystal structure bound to avapritinib (PDB: 8PQH) with the activation loop (AL) of (**D**) WT D842, (**E**) D842V, (**F**) D842I, (**G**) D842A, and (**H**) D842G. In **E** and **F**, the green shading indicates that side chains protrude not only into the hydrophobic back pocket and *stabilize* the active conformation but also fit in a binding pocket formed by the αC-helix and the JMD, whereas in **G** and **H**, the red shading indicates that the alanine side chain or no side chain in case of glycine do not protrude into the same pocket between the αC-helix and the JMD and subsequently less likely stabilizing the activation loop in an active conformation.

Analysis of PDGFRA-WT structure (PDB ID: 8PQJ) revealed that W559 from the JMD inserts into a hydrophobic back pocket, which stabilizes inactive PDGFRA and a DFG-out conformation (Fig. 4A). Upon closer inspection of the activation loop, the WT D842 residue forms a complex interaction network with nitrogen atoms of M844 and H845 and water molecules that were crystallized in the structure (Supplementary Fig. S7A). The stabilization of the inactive conformation allows for the binding of type II TKIs like imatinib (26, 54, 62, 63). When analyzing the structure of PDGFRA-WT bound to imatinib (PDB ID: 6JOL), the kinase maintains a DFG-out conformation, and imatinib displaces W559 from the exact same hydrophobic back pocket (Fig. 4B) while engaging polar interactions with residues E644, V814, H815, and D836, also as previously described (Supplementary Fig. S7B; ref. 61). Imatinib’s polar interactions with D836 are homologous to its interactions with D810 in KIT (54), and together with the interaction with E644, connect the activation loop and further stabilize the inactive kinase conformation (Supplementary Fig. S7C). Despite the JMD displacement, the kinase stays in the DFG-out conformation, with the αC-helix stabilized in the inactive state (Supplementary Fig. S7C).

In contrast to imatinib, type I TKI avapritinib binds to DFG-in, active kinase conformations. To date, the only publicly available PDGFRA crystal structure with avapritinib bound is the PDGFRA-T674I mutant kinase (PDB ID: 8PQH). Examining the structure of the kinase domain of PDGFRA-T674I aligned with apo PDGFRA-WT αC-helix (PDB ID: 8PQJ), we observed that avapritinib occupies the solvent front and Gα pocket (Fig. 4C), in contrast to imatinib’s binding mode, which mainly occupies the hydrophobic back pocket (Fig. 4B). Direct comparison of the structures of PDGFRA-WT bound to imatinib versus PDGFRA-T674I bound to avapritinib showed that imatinib would directly interfere with the DFG motif in an active state (Supplementary Fig. S7D). With the shift to a DFG-in conformation, the activation loop folded into the hydrophobic back pocket, and the F837 of the DFG motif occupied a comparable position of W559 seen in the inactive state (Supplementary Fig. S7D). This further confirmed that the PDGFRA kinase requires an inactive, DFG-out conformation to accommodate imatinib binding into the hydrophobic back pocket.

After confirming imatinib and avapritinib binding states to PDGFRA, we used the PyMOL mutagenesis tool on the kinase domain of the PDGFRA-T674I structure bound to avapritinib (PDB: 8PQH) to model D842A, D842G, D842V, and D842I mutant kinases, always using the most recommended rotamer of the software. As the WT D842 side chain was not resolved in the crystal structure (PDB: 8PQH), we also modeled this in PyMOL for visualization and showed that D842 was repositioned in the active state, binding into a pocket mainly formed by hydrophobic JMD amino acids as well as the αC-helix (Fig. 4D). Notably, this hydrophobic pocket is distinct from the hydrophobic back pocket within the kinase domain and extends toward the “back” of the kinase domain. Our *in silico* modeling showed that the mutation to D842V caused the hydrophobic V residue side chain to protrude into the hydrophobic back pocket and occupied the same position as the JMD residue V561 in the autoinhibited, inactive kinase conformation, mimicking the same structural motif beneath the αC-helix (Fig. 4E). We see this same localization and side-chain protrusion into the hydrophobic back pocket with the D842I mutation as well (Fig. 4F). In contrast, when modeling both D842A and D842G, Ala (A) and Gly (G) side chains did not extend into the hydrophobic back pocket (Fig. 4G and H), leading to increased activation loop flexibility and a much less pronounced interaction within the hydrophobic back pocket, which can accommodate imatinib binding within this pocket. These structural models provided further support for a potential mechanism explaining why Ala 842-position mutations conferred *in vitro* imatinib sensitivity and potentially how 842-position mutations change kinase conformation.

### Applying *in vitro* imatinib IC_50s_ to predict clinical benefit for *PDGFRA* non-D842V, exon 18-mutant GIST

Lastly, to determine whether our cell-based biochemical imatinib sensitivities correlated with clinical response, we analyzed the mPFS probability of 116 patients with *PDGFRA* GIST who were previously treated with first-line imatinib. This cohort of patient response data, listed in Supplementary Table S7, was curated from The Life Raft Group Patient Registry (*n* = 19; ref. 50), previously published EORTC data (*n* = 49; ref. 31), previously published data from a Korean series (*n* = 13; ref. 30), previously published phase II B2222 study (*n* = 5; ref. 20) and unpublished data from European sarcoma centers (*n* = 17), Oregon Health and Science University (*n* = 3), MD Anderson (*n* = 8), and Memorial Sloan Kettering Cancer Center (*n* = 2). Previously published data were used to increase the sample size, as there were few patients with *PDGFRA*-mutant GIST treated with first-line imatinib with associated clinical outcomes from our unpublished sources. *PDGFRA* mutation testing results, first-line therapy information, and time to progression after treatment initiation were used for this retrospective analysis.

**Figure 5.**
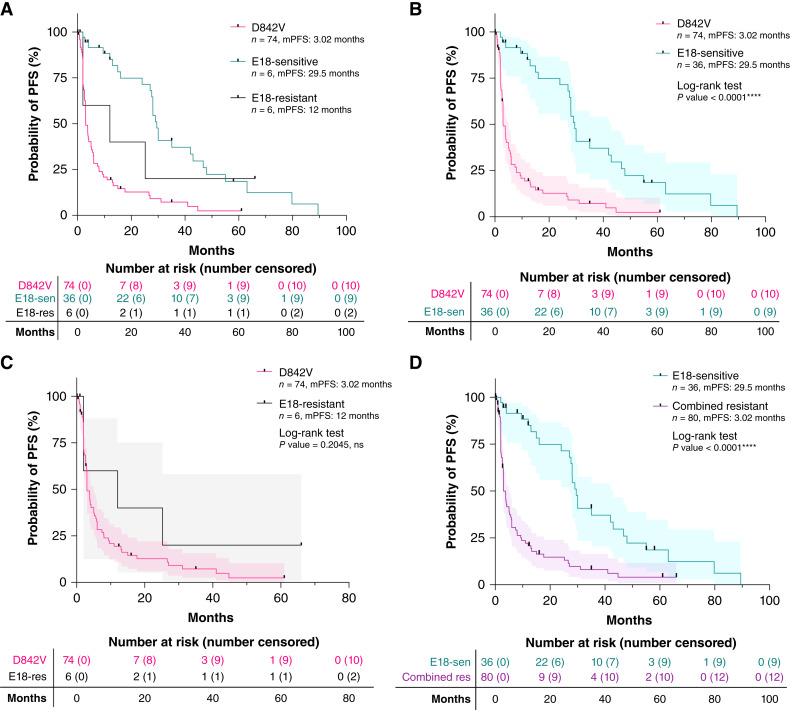
Retrospective analysis of the PFS for 116 patients with *PDGFRA* exon 18–mutant GIST treated with first-line imatinib. The mPFS for patients with* PDGFRA* GIST groups were categorized by their mutation status: D842V, predicted exon 18–sensitive, and predicted exon 18–resistant. Kaplan–Meier survival analysis was used along with log-rank (Mantel–Cox) tests to analyze statistical significance. Risk assessment tables are underneath each graph, displaying the number of patients at risk and, in parentheses, the number censored, for each time point. **A,** The mPFS for patients with D842V (*n* = 74), predicted exon 18–sensitive (*n* = 36), and predicted exon 18–resistant (*n* = 6) mutations. Shaded areas represent the 95% CI of each survival curve. **B,** Comparison of mPFS between D842V and exon 18–sensitive groups (log-rank ****, *P* value < 0.0001). Shaded areas represent the 95% CI of each survival curve. **C,** Comparison of mPFS between D842V and exon 18–resistant groups (log-rank *P* value = 0.2045, ns). Shaded areas represent the 95% CI of each survival curve. **D,** Comparison of mPFS between exon 18–sensitive and combined resistant mutation groups (log-rank ****, *P* value < 0.0001). Shaded areas represent the 95% CI of each survival curve.

We divided patients into four groups based on mutation status: D842V, predicted exon 18–sensitive, predicted non-D842V exon 18–resistant, and a combined resistant group (D842V plus the predicted non-D842V exon 18–resistant group). The predicted sensitive and resistant groups were determined by the amino acid at the 842 position and corresponding *in vitro* classifications from Fig. 3. Mutations with 842-position amino acids with intermediate resistance were placed in the exon 18–resistant group. The mPFS for D842V patients (*n* = 74) treated with first-line imatinib was 3.02 months (95% CI, 2–5 months; Fig. 5A), consistent with prior reports (9, 30–32). In comparison, the mPFS for patients with predicted exon 18–resistant mutations (*n* = 6) was 12 months (95% CI, 2 to infinity months), whereas for those with predicted exon 18–sensitive mutations (*n* = 36), the mPFS was 29.5 months (95% CI, 27–42.97 months; Fig. 5A). Statistical analysis showed a significant difference between the mPFS for D842V and exon 18–sensitive mutations (3.02 months vs. 29.5 months, log-rank test, *P* value < 0.0001, Fig. 5B) but no significant difference between the mPFS for D842V and exon 18–resistant mutant patients (log-rank test, *P* value = 0.2045, Fig. 5C). As our ultimate goal was to determine whether cases from our series with predicted exon 18–sensitive mutations were concordant with clinical outcomes, we compared the mPFS of patients with predicted exon 18–sensitive mutations versus those patients in the combined resistant mutation group (D842V + predicted exon 18–resistant). There was a significant difference between the mPFS of these groups (29.5 months vs. 3.02 months, log-rank test, *P* value < 0.0001; Fig. 5D). In summary, patients with predicted exon 18–sensitive disease treated with first-line imatinib experienced a much longer mPFS than those with D842V, and other predicted exon 18–resistant mutant GISTs, supporting the notion that our predictive *in vitro* IC_50_ values of 842-position amino acid mutations correlate with clinical outcomes.

## Discussion

In this study, we provide paradigm-shifting evidence to optimize the treatment of *PDGFRA*-mutant GIST based on our predictive model systems. The use of targeted TKIs like imatinib for GIST, a previously untreatable disease, is a prime example of how precision oncology transforms patient care. First-line imatinib is currently estimated to confer a mPFS of 39.4 months for those with *KIT*-mutant GIST, with a median overall survival that has not been reached but is 69% at 5 years (64). However, patients with the most common type of *PDGFRA*-mutant GIST, exon 18 D842V, display primary clinical resistance to imatinib with an overall response rate (ORR) of <10% and a mPFS of <4 months (30, 31). Patients with *PDGFRA* D842V-mutant GIST also do not benefit from other approved GIST type II TKIs due to how this mutation alters the kinase to adopt a structure that interferes with type II TKI binding. However, the rational design of avapritinib, a type I TKI, to target D842V specifically, led to its outstanding clinical efficacy for patients with D842V-mutant GIST, evident by a high ORR of 91% and a mPFS of 34 months (28, 33, 34, 65). Avapritinib is currently FDA-approved for first-line treatment of all *PDGFRA* exon 18–mutant GIST, whereas other international regulatory health authorities restrict usage to *PDGFRA* D842V-mutant GIST only (38, 66). Although we know that D842V-mutant GIST requires avapritinib treatment, previously, it was largely unknown whether other exon 18 mutations require this treatment. Although there are some sporadic clinical reports that some exon 18 mutant–GISTs respond to imatinib, evidence-based predictions on which types of observed mutations would be imatinib sensitive were limited prior to our study. Our goal was to identify clinical cases in which imatinib can be used as first-line treatment for non-D842V, *PDGFRA* exon 18–mutant GIST and provide evidence supporting the use of imatinib as a viable alternative treatment option for those unable to receive or tolerate avapritinib.

Previously, there were no comprehensive published databases of the mutations observed in *PDGFRA*-mutant GIST, as many institutions do not report their cases to public databases. Our assembled database of *PDGFRA*-mutant GIST presented in this study is the largest reported to date. Among our 1,000 cases, many unique non-D842V exon 18 *PDGFRA* mutations were observed; thus, it was not feasible to model every mutation to determine TKI sensitivity. However, nearly 78% of all observed exon 18 mutations directly involved the 842 residue. This WT D842 residue in the PDGFRA activation loop plays an important role in autoinhibition of the kinase and maintains key interactions within the activation loop that stabilize a DFG-out, inactive state (28). Notably, D842V destabilizes an inactive kinase conformation, which leads to imatinib resistance, highlighting an important relationship between mutation-induced structural changes and imatinib sensitivity. In our cohort, we observed nearly every amino acid change at the 842-position, therefore we speculated that these other mutations may affect the function of the key autoinhibitory 842-position residue. We hypothesized that the chemical characteristics of amino acids at the 842-position play a direct role in determining imatinib sensitivity or resistance and that modeling all possible mutations at this position could yield predictive results applicable to all present and future cases of *PDGFRA* exon 18 mutations. This investigation is translatable to other related class III RTKs like KIT and FLT3, as these also have a homologous autoinhibitory residue in the activation loop (KIT D816, FLT3 D835) that is frequently altered in other cancer types that are treated with TKIs.

**Figure 6.**
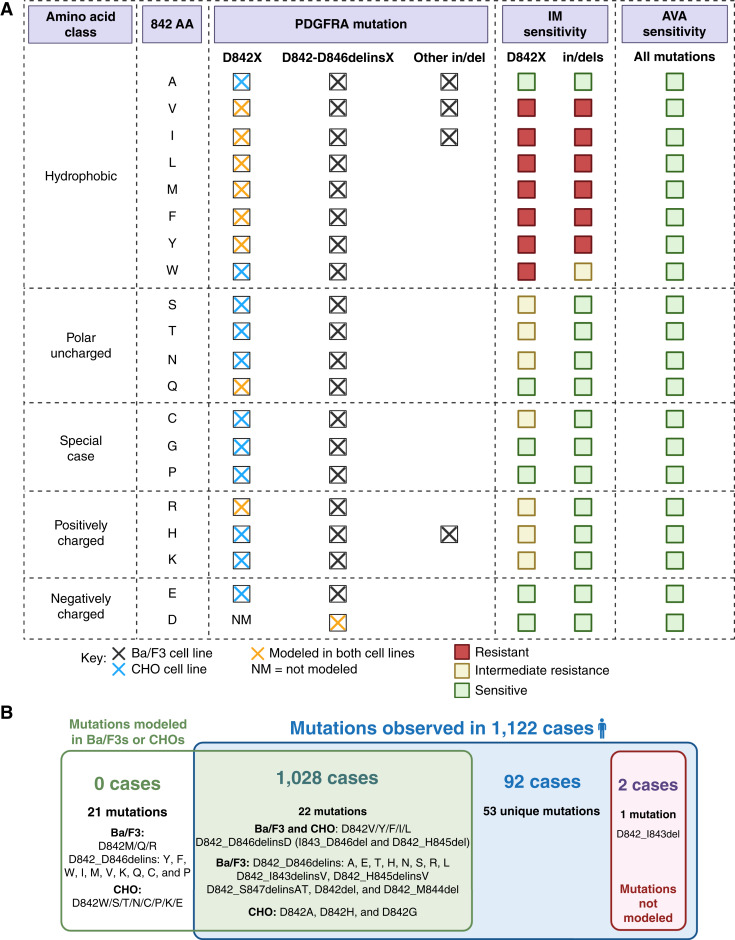
*PDGFRA* exon 18 mutations modeled over the course of the study, with their respective TKI sensitivities and representation within the cases in the cohort. **A,** Summary of Ba/F3 and CHO *in vitro* imatinib (IM) and avapritinib (AVA) sensitivities based on the 842 amino acid (AA) at the exon 18, 842-position. Colored Xs in boxes represent the PDGFRA D842X, D842_D846delinsX, or other in/del mutations that were modeled by a specific cell line (black, Ba/F3; blue, CHO; and orange, both). NM refers to a mutation that was not modeled in any cell line. Drug sensitivities in the far-right columns are based on the IC_50_ data shown in Fig. 3. **B,** Venn diagram showing the overlap between the *PDGFRA* mutations modeled in this study compared with the mutations observed in the exon 18 patient cohort. The green box represents mutations that were successfully modeled and tested in Ba/F3s and/or CHOs, specifying which mutations were modeled in which cell line. The blue box represents the mutations observed in our cohort of 1,122 cases in total. The red box represents the mutations that were not transformed in Ba/F3s and were not modeled in CHOs. Large numbers within each colored box represent the number of cases from the patient cohort that fall into each category within the Venn diagram. [Created in BioRender. Khosroyani, H. (2026) https://BioRender.com/bj6mcai.]

We determined that the amino acid at the 842 position determined imatinib sensitivity and the number of deleted residues in the final protein did not significantly alter sensitivity trends (Fig. 6A). In some cases, deleted residues around the 842 position seemed to sensitize the mutant kinase, as full-length D842X mutations with S, T, N, C, R, H, and K exhibited intermediate resistance but were imatinib-sensitive in the D842_D846delinsX context. Although these differences could be attributed to the specific model we used (CHO vs. Ba/F3s), we speculate that deleted residues around this position could offer changes in kinase structure that slightly increase imatinib binding affinity; however, additional studies are needed to investigate this.

Also demonstrated in our study, we know that imatinib cannot be used as an alternative treatment option for all non-D842V exon 18–mutant GISTs, as clinically reported D842Y and D842I mutations both display *in vitro* imatinib resistance (29) and most likely require avapritinib treatment. As avapritinib is currently unavailable outside the United States for use in non-D842V settings, these patients with type II TKI-resistant *PDGFRA*-mutant GISTs are still left with no effective treatment options. With our first comprehensive report on the avapritinib sensitivities for different exon 18 mutations, we show that avapritinib could be used to treat any 842-position mutation regardless of imatinib sensitivity or resistance. Our data provide evidence that the usage of avapritinib should be expanded to patients with non-D842V cases that have predicted imatinib-resistant mutations. Over the course of the study, we created cell lines with the exact mutation seen in 1,028 of 1,122 total exon 18 cases (approximately 92%, Fig. 6B). Although we also profiled mutations not seen in our clinical cases, if these mutations were to be observed in future GISTs or other *PDGFRA*-mutant driven diseases, we provide the first report of their imatinib and avapritinib sensitivities that can be used to determine treatment options.

More than 80% of cases in our *PDGFRA*-mutant GIST cohort had exon 18 mutations, which affect the activation loop. Activation loop flexibility plays a critical role in promoting an active state and the binding of substrates, whereas the orientation of structural elements, such as the DFG motif, influences imatinib binding (51, 56, 63, 67). Therefore, our biochemical imatinib IC_50_ data imply how different mutations at the 842-position may influence the orientation of the DFG and induce a specific kinase conformation. Our *in silico* modeling demonstrated that imatinib-sensitive D842A and D842G mutations lead to a less stabilized active conformation due to a far less pronounced side-chain protrusion in the hydrophobic pocket, allowing for the adoption of a structure that can more readily accommodate imatinib binding. This suggests that other imatinib-sensitive mutations may use similar structural characteristics and effects on the activation loop and most likely remain in a conformation that allows for imatinib binding. Although these *in silico* models remain to be verified by *in vitro* crystallization, nuclear magnetic resonance, or cryo-electron microscopy studies, they provide a plausible mechanistic explanation for how mutations at the 842-position influence type II–specific TKI binding.

When correlating our *in vitro* IC_50_ value predictions to first-line imatinib treatment responses in patients with *PDGFRA*-mutant GIST, there was a strong concordance between our biochemical results and clinical outcomes. The mPFS for patients with *PDGFRA* D842V versus predicted exon 18 imatinib-sensitive tumors were 3.02 and 29.5 months, respectively (*P* < 0.0001). There was also no significant difference in the mPFS of patients with predicted exon 18–resistant mutations and those with D842V tumors (*P* = 0.2045). However, a limitation of our analysis is the small sample size of patients with non-D842V but a predicted exon 18–resistant mutation (*n* = 6). Despite these limitations, as patients with predicted imatinib-sensitive mutations experienced a longer mPFS than those with predicted imatinib-resistant or D842V mutations, our biochemical results should still be used as a reference for making clinical decisions regarding the treatment for any current or future observed *PDGFRA* exon 18 mutation. As more eligible patients are treated with first-line imatinib, clinical response data should be collected to further strengthen our predictions. Physicians should consider best initial treatment, especially in situations in which it is critical to induce a rapid clinical response due to the risk of adverse outcomes if tumor control is not immediately obtained with the first-line therapy. Until our predictive results are further validated by more clinical data, avapritinib should absolutely be considered as the first option in life-threatening cases, if available to the patient, even in cases with predicted imatinib-sensitive disease.

Patients with advanced, unresectable GIST typically require chronic TKI treatment to achieve disease control, and unfortunately, many develop secondary resistance mutations over time, which lead to disease progression (68). Unfortunately, those who develop avapritinib resistance do not have any approved second-line therapy to treat their resistant tumors (13, 37). However, those who receive imatinib first may have additional salvage therapies to treat their progressive disease if they develop secondary mutations. We anticipate that patients with *PDGFRA*-mutant GIST with predicted imatinib-sensitive tumors that are treated with imatinib will eventually develop resistance mutations analogous to those previously observed in imatinib-resistant, *KIT*-mutant GIST. As most patients with *PDGFRA*-mutant GIST have imatinib-resistant D842V tumors, most studies of secondary resistance in *PDGFRA*-mutant GIST are in the context of avapritinib resistance. Our knowledge about the mutational landscape of secondary imatinib resistance in non-D842V, exon 18 *PDGFRA*-mutant GIST is limited and warrants further investigation as we begin to use imatinib to intentionally treat more patients. Subsequent studies on secondary imatinib resistance will provide insight into additional treatment options that will inform further treatment decisions after progression.

Individually creating models of patient-associated mutations as they are observed is not feasible and is time-consuming. The development of our predictive *in vitro* models provided a framework for us to comprehensively profile exon 18 mutations and their imatinib sensitivity, while also identifying patients with tumors that would require avapritinib treatment. Overall, our data highlight the importance of understanding the molecular mechanisms by which these mutations induce TKI sensitivity or resistance. Our approach further optimizes treatment options for *PDGFRA* exon 18–mutant GIST, thereby improving clinical outcomes by matching patients with the most suitable and accessible first-line TKI.

## Supplementary Material

Supp. Fig. 1 Supplementary Figure 1

Supp. Fig. 2 Supplementary Figure 2

Supp. Fig. 3 Supplementary Figure 3

Supp. Fig. 4 Supplementary Figure 4

Supp. Fig. 5 Supplementary Figure 5

Supp. Fig. 6 Supplementary Figure 6

Supp. Fig. 7 Supplementary Figure 7

Supplementary Table S1 Table S1. List of oligo sequences for each *PDGFRA* mutation modeled successfully in either Ba/F3s or CHOs.

Supplementary Table S2 Table S2. List of primer sequences used to PCR amplify and sequence *PDGFRA* cDNA.

Supplementary Table S3 Table S3. List of primary mutations seen in our 1379 *PDGFRA*-mutant GIST case cohort. Mutations are separated out by exon region in which they are found.

Supplementary Table S4 Table S4. Imatinib IC_50_ values and 95% confidence intervals (CIs) of *PDGFRA *mutations modeled in Ba/F3 and CHO cells.

Supplementary Table S5 Table S5. Avapritinib IC_50_ values and 95% confidence intervals (CIs) of *PDGFRA *mutations modeled in Ba/F3 and CHO cells.

Supplementary Table S6 Table S6. List of *PDGFRA* mutations that were observed in the cases within our cohort, along with information about whether these mutations were modeled in vitro.

Supplementary Table S7 Table S7. Clinical responses of advanced *PDGFRA*-mutant GIST patients treated with first-line imatinib.

## Data Availability

All analyzed data generated for this study are included within the article and corresponding supplementary data files. Raw data are available upon request to the corresponding author.
