# Supplementary material for: Predicting Clinical Sensitivities of PDGFRA Exon 18 Mutations to Imatinib and Avapritinib to Optimize Gastrointestinal Stromal Tumor Treatment
Source: Cancer Res Commun. 2026 Jul 6;6(7):1573–91. doi: 10.1158/2767-9764.CRC-26-0093 (PMC13333789; doi:10.1158/2767-9764.CRC-26-0093)
Supplement: Supp. Fig. 1 — Supplementary Figure 1 [file crc-26-0093_supp.fig.1_suppsf1.pdf]

Supp. Fig. 1

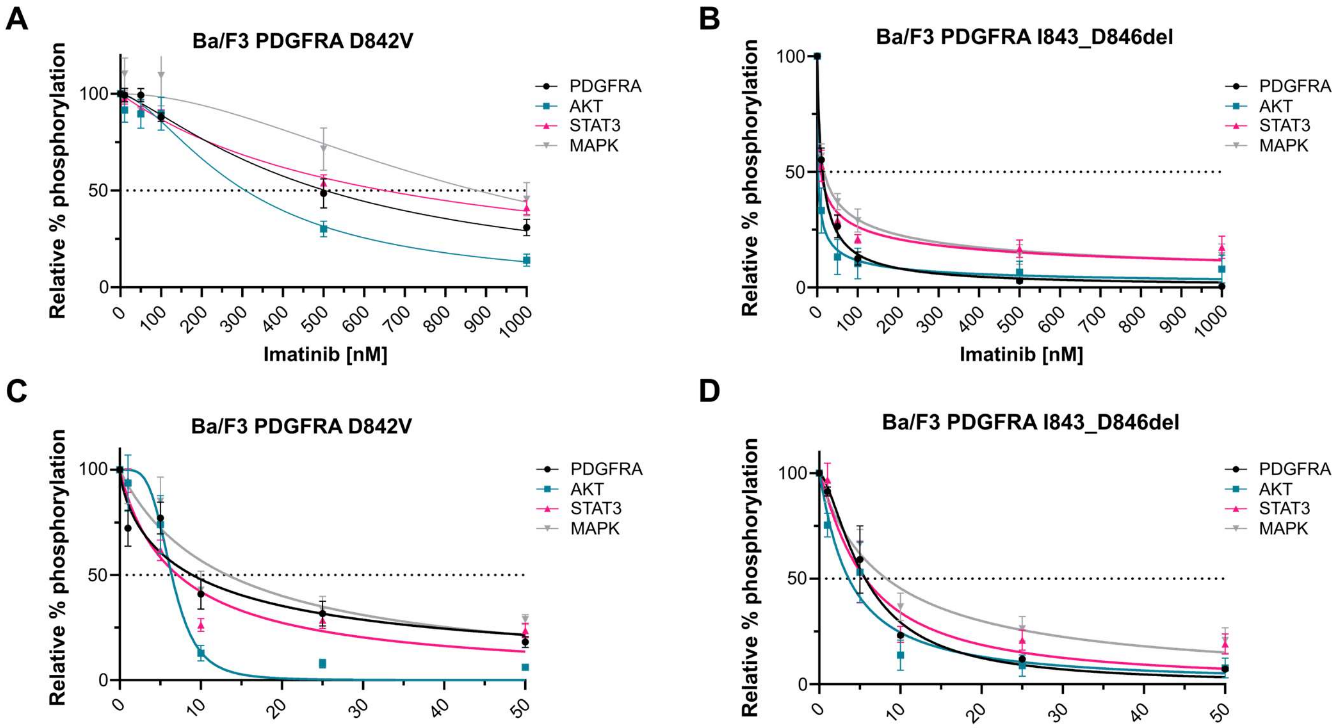

Supp. Fig. 1: Immunoblot quantification of the effects of imatinib and avapritinib treatment on the inhibition of phosphorylated-PDGFRA and downstream signaling effector molecules in Ba/F3 PDGFRA D842V and I843_D846del cell lines. Lysates were harvested after 90 minutes of exposure to varying doses of imatinib (IM) and avapritinib (AVA), and equal amounts of protein were loaded for each tested dose. Data presented were derived from at least 3 independent experiments. Representative immunoblots are shown in Figure 2 within the main manuscript. Quantifications of the relative % phosphorylation determined by densitometry ratios of phosphorylated- to total-protein isoforms for PDGFRA, AKT, STAT3, and MAPK for **A)** Ba/F3 D842V cells and **B)** Ba/F3 I843_D846del cells treated with imatinib. Non-linear regression analysis was performed in GraphPad Prism, and a horizontal dotted line is marked at 50%, with interceptions to depict the concentration of the drug IC_{50}. Quantifications of the relative % phosphorylation determined by densitometry ratios of phosphorylated- to total-protein for PDGFRA, AKT, STAT3, and MAPK for **C)** Ba/F3 D842V cells and **D)** Ba/F3 I843_D846del cells treated with avapritinib. Analyses and graphical representation are the same as in panels A & B.
