# Supplementary material for: Predicting Clinical Sensitivities of PDGFRA Exon 18 Mutations to Imatinib and Avapritinib to Optimize Gastrointestinal Stromal Tumor Treatment
Source: Cancer Res Commun. 2026 Jul 6;6(7):1573–91. doi: 10.1158/2767-9764.CRC-26-0093 (PMC13333789; doi:10.1158/2767-9764.CRC-26-0093)
Supplement: Supp. Fig. 2 — Supplementary Figure 2 [file crc-26-0093_supp.fig.2_suppsf2.pdf]

Supp. Fig. 2

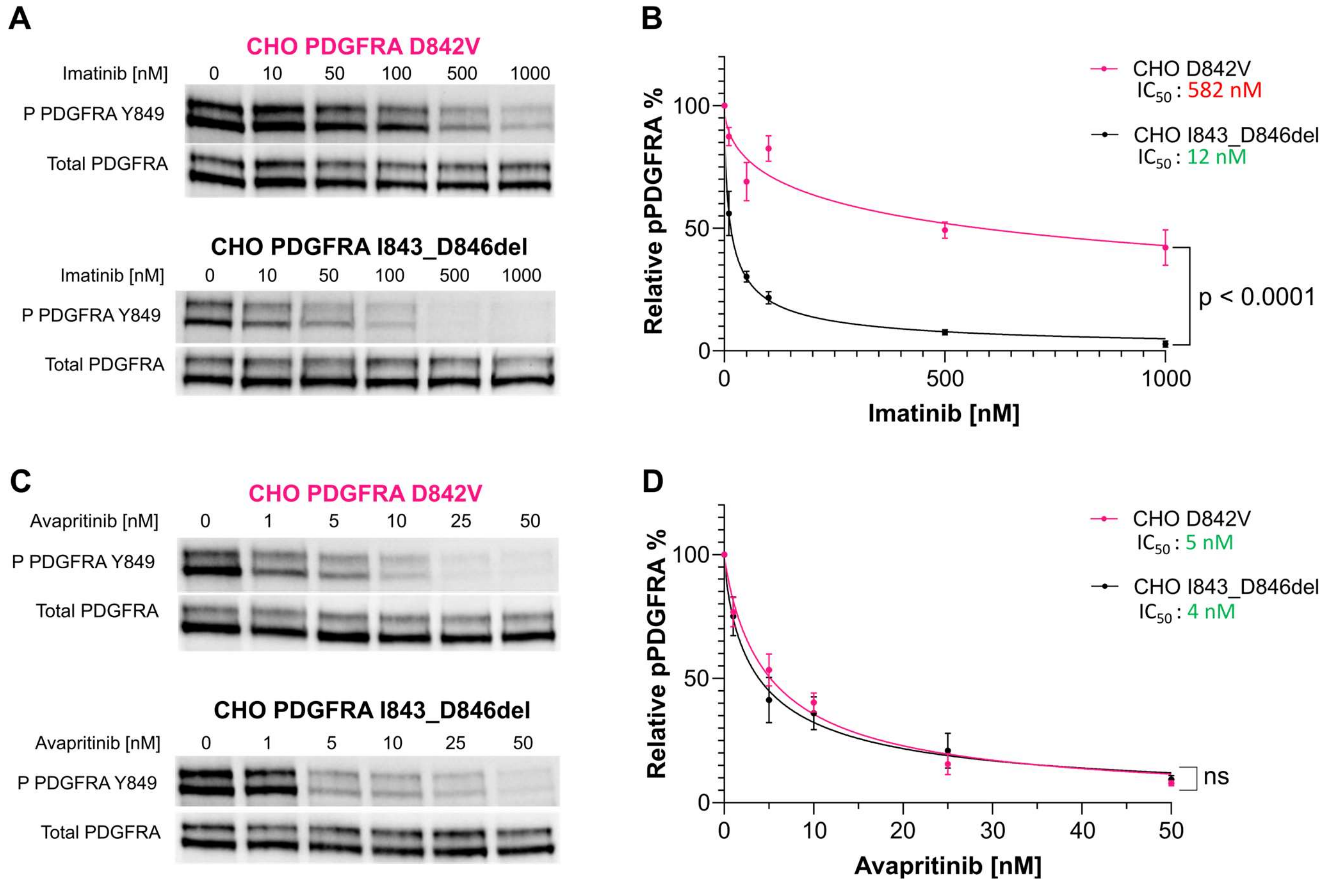

Supp. Fig. 2: CHO PDGFRA D842V and PDGFRA I843_D846del cells recapitulate clinical imatinib and avapritinib sensitivities. The effect of imatinib and avapritinib on the phosphorylation of PDGFRA was used to calculate drug IC_{50s} using non-linear regression in GraphPad Prism. **A)** Representative immunoblot image of stably expressing PDGFRA D842V and PDGFRA I843_D846del mutations in CHO cells treated with various doses of imatinib. Phosphorylated PDGFRA and total PDGFRA are shown. **B)** Calculated imatinib IC_{50s} for CHO PDGFRA D842V and CHO PDGFRA I843_D846del cells, data represented are from at least three independent experiments, with error bars representing the SEM. The p-value corresponds to the result from an extra sum of squares F test, indicating that the difference between the IC_{50} values/non-linear regression curves was statistically significant. **C)** Representative immunoblot images of CHO cells stably expressing PDGFRA D842V or PDGFRA I843_D846del cells treated with various doses of avapritinib. Phosphorylated PDGFRA and total PDGFRA are shown. **D)** Calculated avapritinib IC_{50s} for CHO PDGFRA D842V and CHO PDGFRA I843_D846del cells, with data from at least three independent experiments and error bars representing the SEM. The p-value corresponds to the result from an extra sum of squares F test, indicating that the difference between the IC_{50} values/non-linear regression curves was not significant.
