# Supplementary material for: Predicting Clinical Sensitivities of PDGFRA Exon 18 Mutations to Imatinib and Avapritinib to Optimize Gastrointestinal Stromal Tumor Treatment
Source: Cancer Res Commun. 2026 Jul 6;6(7):1573–91. doi: 10.1158/2767-9764.CRC-26-0093 (PMC13333789; doi:10.1158/2767-9764.CRC-26-0093)
Supplement: Supp. Fig. 3 — Supplementary Figure 3 [file crc-26-0093_supp.fig.3_suppsf3.pdf]

Supp. Fig. 3

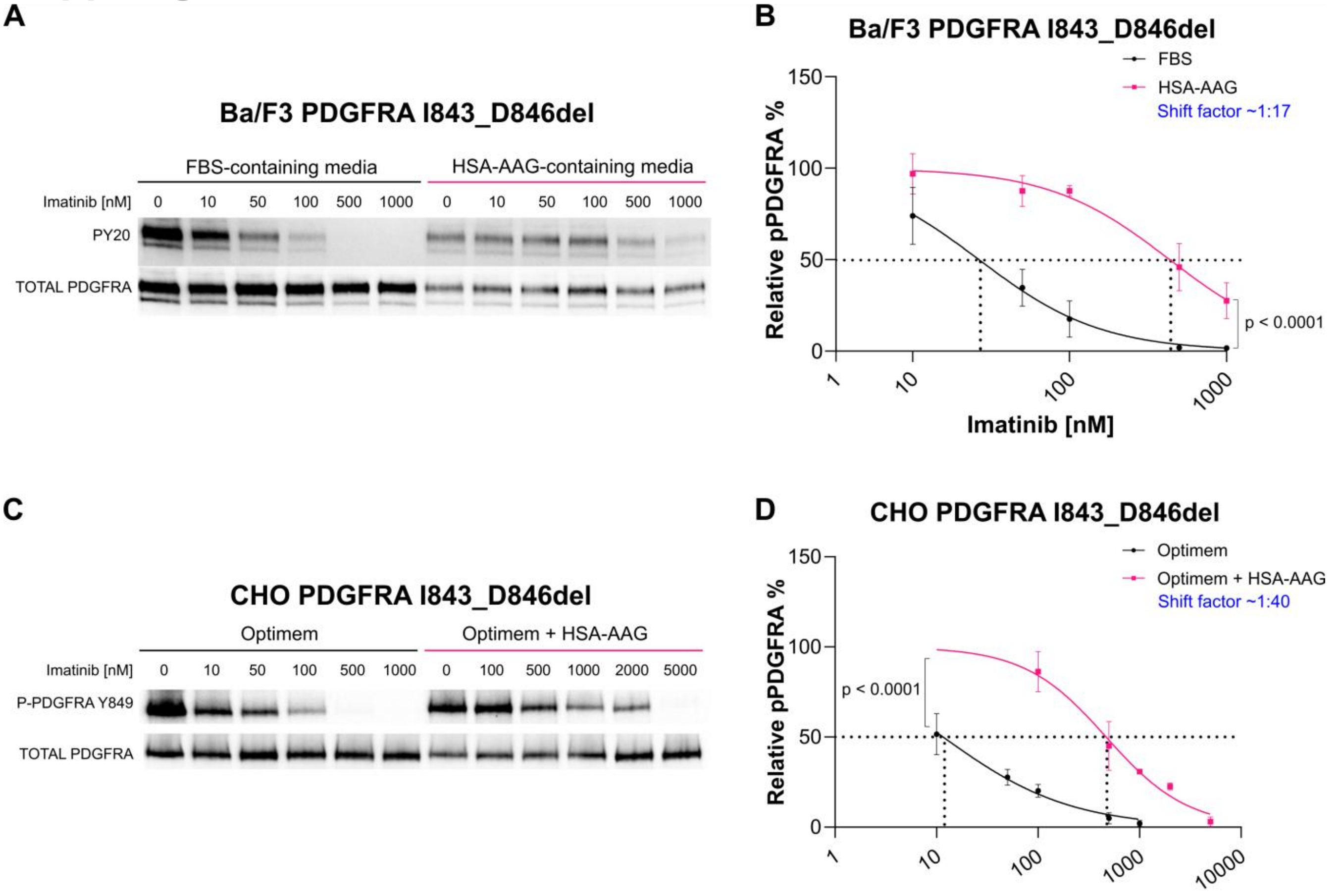

Supp. Fig. 3: The effects of human serum proteins (HSA-AAG) on calculated imatinib IC_{50} values in Ba/F3 and CHO models. A) Representative image from immunoblotting experiments testing the inhibitory effects of imatinib in Ba/F3 PDGFRA I843_D846del cells grown in media with FBS or HSA-AAG. PY20 refers to phosphorylated-tyrosine at 190 kDa, which corresponds to phosphorylated-PDGFRA. **B**) Plotted is the relative percentage of phosphorylated-PDGFRA calculated with densitometry using the ratio of phosphorylated- to total-PDGFRA relative to Ba/F3 PDGFRA I843_D846del cells without imatinib treatment. Data points represent the average ± SEM of at least 3 independent experiments. The y-axis dotted line indicates where 50% inhibition occurs, corresponding to the IC_{50} value. Vertical x-axis dotted lines indicate the drug dose where 50% inhibition of phosphorylated-PDGFRA occurred. The p-value corresponds to the result from an extra sum of squares F test, indicating that the difference between the IC_{50} values/non-linear regression curves was statistically significant. **C)** Representative image from immunoblotting experiments testing the inhibitory effects of imatinib in CHO PDGFRA I843_D846del cells grown in Optimem only or Optimem + HSA-AAG media. **D)** Plotted is the relative percentage of phosphorylated-PDGFRA calculated with densitometry using the ratio of

phosphorylated- to total-PDGFRA relative to cells without imatinib treatment. Data points represent the average ± SEM of at least 3 independent experiments. The y-axis dotted line indicates where 50% inhibition occurs, corresponding to the IC_{50} value. Vertical x-axis dotted lines indicate the drug dose where 50% inhibition of phosphorylated-PDGFRA occurred. The p-value corresponds to the result from an extra sum of squares F test, indicating that the difference between the IC_{50} values/non-linear regression curves was statistically significant.
