# Supplementary material for: Predicting Clinical Sensitivities of PDGFRA Exon 18 Mutations to Imatinib and Avapritinib to Optimize Gastrointestinal Stromal Tumor Treatment
Source: Cancer Res Commun. 2026 Jul 6;6(7):1573–91. doi: 10.1158/2767-9764.CRC-26-0093 (PMC13333789; doi:10.1158/2767-9764.CRC-26-0093)
Supplement: Supp. Fig. 4 — Supplementary Figure 4 [file crc-26-0093_supp.fig.4_suppsf4.pdf]

Supp. Fig. 4

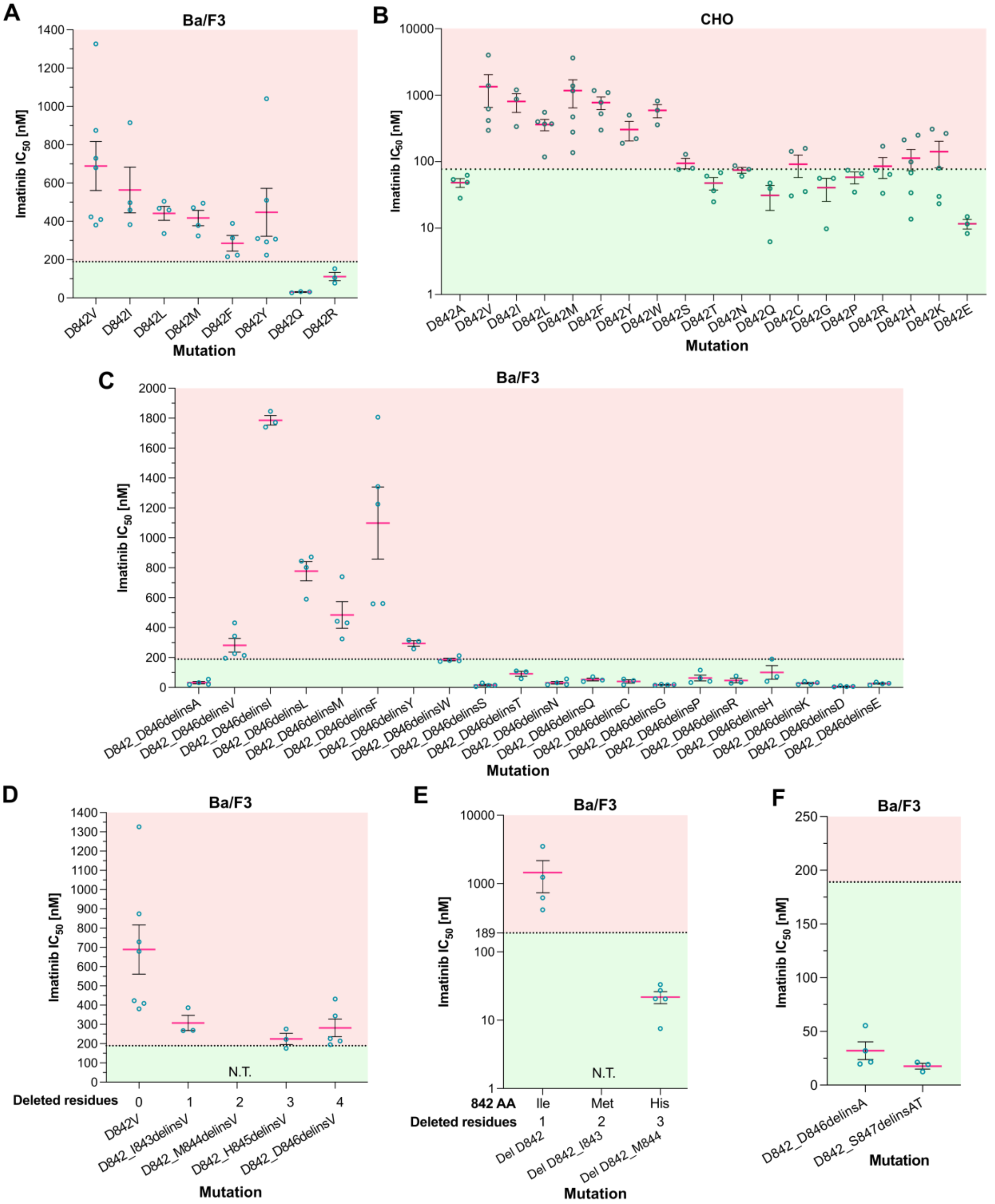

Supp. Fig. 4: Mean imatinib IC50s of transforming Ba/F3 and stably expressing CHO PDGFRA 842-position mutation cell lines. Immunoblot quantification of the effect of imatinib on the phosphorylation of PDGFRA in **A**) Ba/F3 D842X cell lines, **B**) CHO D842X cell lines, **C)** Ba/F3 842 4-residue deletion mutation cell lines, and **D-F**) additional deletion mutations profiled

in Ba/F3s. Whole cell lysates were harvested after 90 minutes of exposure to imatinib at various doses. Equal amounts of lysates were loaded for all samples, and densitometry was used to calculate the ratio of phosphorylated-PDGFRA to total PDGFRA for each dose. IC_{50s} were calculated using non-linear regression analyses in GraphPad Prism. Each data point in these panels represents the calculated IC_{50} for one given experiment, the pink line indicates the value of the mean IC_{50}, and the error bars are ± SEM. Horizontal dotted lines in **A** and **C-F** are the threshold IC_{50} at 189nM, and in **B**, the threshold IC_{50} is at 77nM. These threshold IC_{50s} were calculated by a serum shift assay, as outlined in the methods, and used to separate predicted clinical resistance versus clinical sensitivity. The red area shaded above this threshold line represents predicted clinical resistance; the green area shaded below this threshold line represents predicted clinical sensitivity. SEMs and data points that cross both the red and green shaded areas are classified with intermediate resistance. “N.T” indicates a non-transforming mutation in the Ba/F3 model system.
