# Supplementary material for: Predicting Clinical Sensitivities of PDGFRA Exon 18 Mutations to Imatinib and Avapritinib to Optimize Gastrointestinal Stromal Tumor Treatment
Source: Cancer Res Commun. 2026 Jul 6;6(7):1573–91. doi: 10.1158/2767-9764.CRC-26-0093 (PMC13333789; doi:10.1158/2767-9764.CRC-26-0093)
Supplement: Supp. Fig. 5 — Supplementary Figure 5 [file crc-26-0093_supp.fig.5_suppsf5.pdf]

Supp. Fig. 5

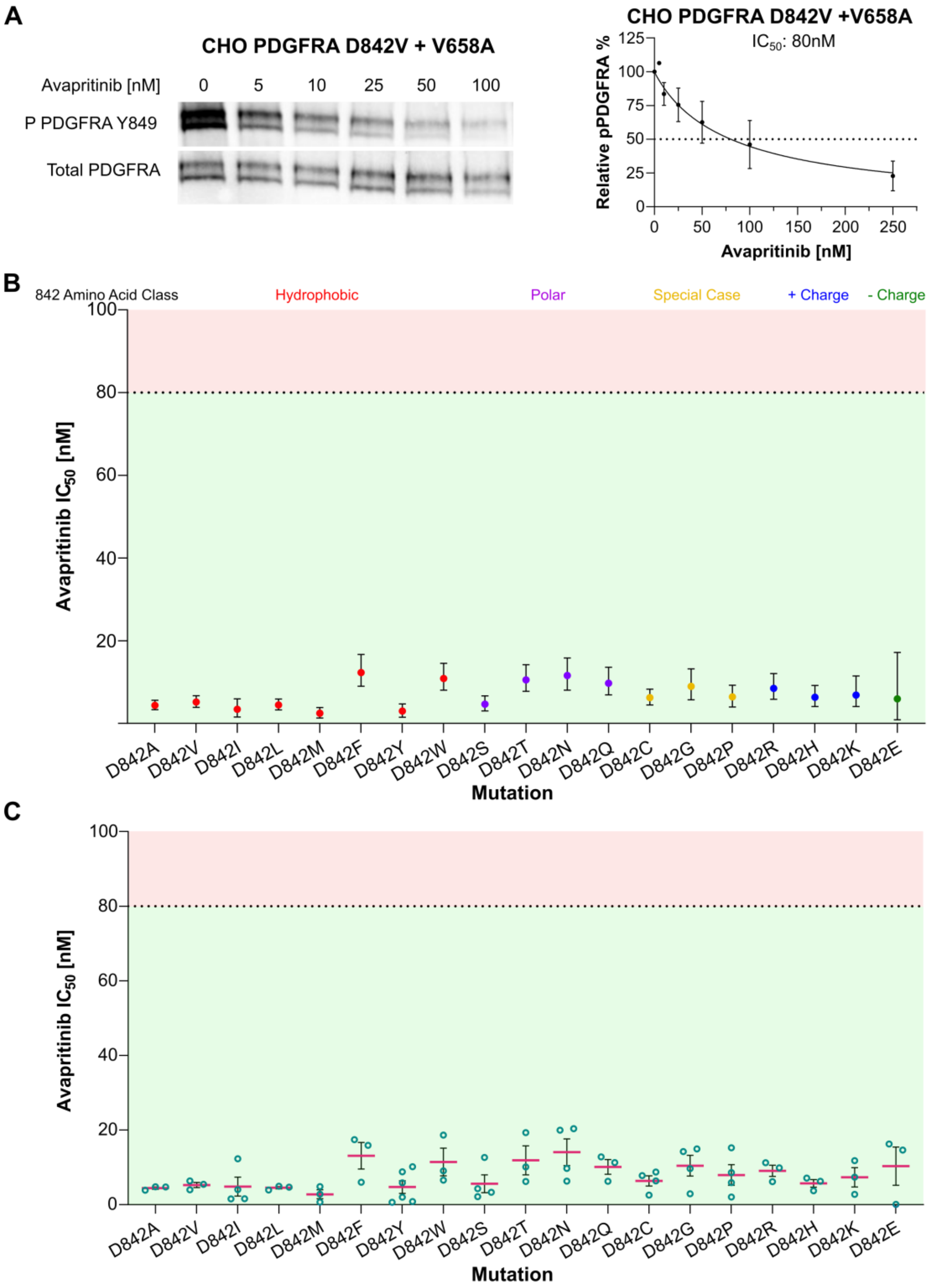

Supp. Fig. 5: Profiling avapritinib sensitivities of CHO PDGFRA D842X mutations using immunoblotting. A) Representative image of immunoblotting experiments testing avapritinib inhibition on phosphorylated-PDGFRA in a stably expressing CHO PDGFRA D842V + V658A cell line, the same

mutation found in a patient from a published study who progressed on avapritinib. The graph represents the dose-response curve of avapritinib on the relative phosphorylated-PDGFRA % from 4 independent experiments, with error bars representing ± SEM and a horizontal dotted line set at 50% inhibition. The calculated IC_{50} value of 80nM is used as the threshold line in **B-C** to determine predicted clinical resistance and sensitivity. **B**) Avapritinib IC_{50} values of CHO D842X mutations plotted with error bars representing ± 95% CIs. Colors of the dots represent the amino acid class. **C)** Same data graphed in B, with each data point a calculated IC_{50} value for one independent experiment, with the pink line indicating the value of the mean IC_{50}, and error bars ± SEM. For all, whole cell lysates were harvested after 90 minutes of exposure to avapritinib at various doses. Same amount of lysate loaded across all samples; densitometry was used to calculate the ratio of phosphorylated-PDGFRA to total PDGFRA across doses. Relative IC_{50} values were calculated using non-linear regression analyses in GraphPad Prism. In **B-C**, the red area shaded above the horizontal threshold line represents predicted clinical resistance, green area shaded below the threshold line represents predicted clinical sensitivity.
