# Supplementary material for: Predicting Clinical Sensitivities of PDGFRA Exon 18 Mutations to Imatinib and Avapritinib to Optimize Gastrointestinal Stromal Tumor Treatment
Source: Cancer Res Commun. 2026 Jul 6;6(7):1573–91. doi: 10.1158/2767-9764.CRC-26-0093 (PMC13333789; doi:10.1158/2767-9764.CRC-26-0093)
Supplement: Supp. Fig. 6 — Supplementary Figure 6 [file crc-26-0093_supp.fig.6_suppsf6.pdf]

Supp. Fig. 6

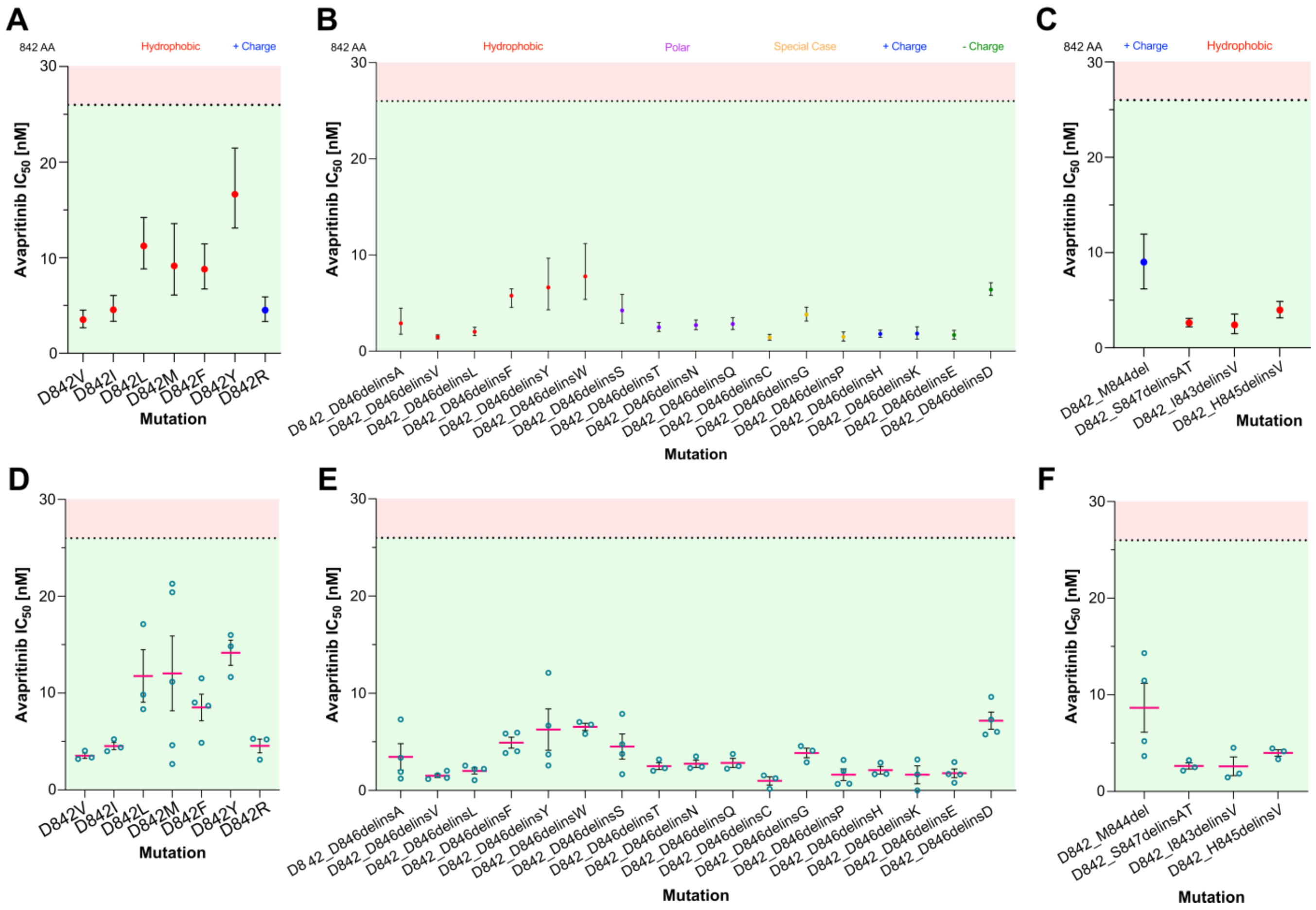

Supp. Fig. 6: Profiling avapritinib sensitivities of transforming Ba/F3 PDGFRA 842-position mutations using immunoblotting. Immunoblot quantification of the effect of avapritinib on phosphorylated-PDGFRA in Ba/F3 cell lines with **A**) D842X point mutations and **B**) 842-position 4-residue deletion mutations, and **C**) additional 842-position deletion mutations. In **A-C**, the data points represent the IC_{50} calculation of at least 3 independent experiments, and error bars indicate the range of the 95% CI for a calculated IC_{50}. The color of the dots represents the class of the 842-position amino acid (842 AA). In **D-F**, the same data is represented (**D-** D842X point mutations, **E-** 842-position 4-residue deletion mutations, **F-** additional 842-position deletion mutations) as each data point is a calculated IC_{50} for one independent experiment, the pink line indicates the value of the mean IC_{50}, and the error bars are ± SEM. For all, whole cell lysates were harvested after 90 minutes of exposure to avapritinib at various doses. Same amount of lysate loaded across all samples; densitometry was used to calculate the ratio of phosphorylated-PDGFRA to total PDGFRA across doses. IC_{50s} were calculated using non-linear regression analyses in GraphPad Prism. Horizontal threshold lines are set at an IC_{50} of 26nM, which was determined by a previously published Ba/F3 PDGFRA D842V + V658A cell line that was modelled after

a patient who progressed on avapritinib treatment. The red area shaded above this threshold line represents predicted clinical resistance, and with green area shaded below this threshold line represents predicted clinical sensitivity.
