# Supplementary material for: Predicting Clinical Sensitivities of PDGFRA Exon 18 Mutations to Imatinib and Avapritinib to Optimize Gastrointestinal Stromal Tumor Treatment
Source: Cancer Res Commun. 2026 Jul 6;6(7):1573–91. doi: 10.1158/2767-9764.CRC-26-0093 (PMC13333789; doi:10.1158/2767-9764.CRC-26-0093)
Supplement: Supp. Fig. 7 — Supplementary Figure 7 [file crc-26-0093_supp.fig.7_suppsf7.pdf]

Supp. Fig. 7

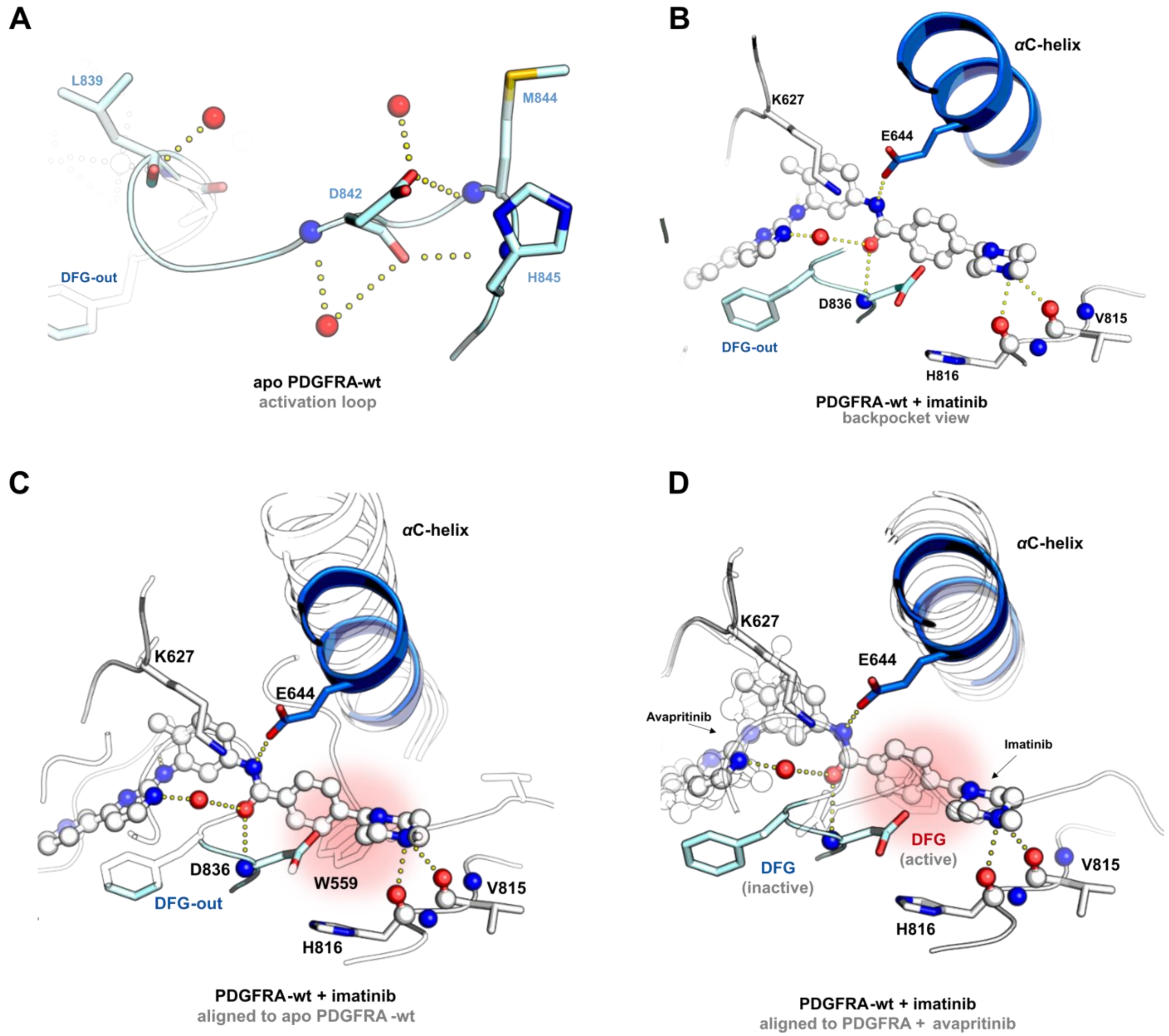

Supp. Fig. 7: Structural visualization of TKI drug binding and interactions with PDGFRA. All visualizations were done in PyMOL. **A)** Visualization of apo-PDGFRA wildtype (wt) (PDB: 8PQJ), highlighting the interactions (indicated by the dotted yellow lines) between wildtype D842 and other amino acids located in the activation loop in the inactive DFG-out conformation. Floating red dots indicate water molecules crystallized within the structure. **B)** Visualization of imatinib bound to PDGFRA-wildtype (PDB: 6JOL) from the back pocket view, showing polar interactions between the drug and amino acid side chains of PDGFRA that stabilize the ligand in the back pocket, indicated by the dotted yellow lines. **C)** Visualization of imatinib bound to PDGFRA-wildtype (PDB: 6JOL) aligned with apo-PDGFRA wildtype (PDB: 8PQJ, not bound to imatinib), showing the displacement of the JMD away from the hydrophobic

back pocket. The red shaded area indicates the region where the W559 residue of the JMD occupies the hydrophobic back pocket when not bound to imatinib. **D)** Visualization of the activation loop of PDGFRA-wildtype bound to imatinib (PDB: 6JOL) aligned with PDGFRA-T674I bound to avapritinib (PDB: 8PQH). The red shaded area indicates the position of the DFG-motif when the kinase is in the active form. Avapritinib binds in a different orientation and not to the hydrophobic back pocket like imatinib.
