# Supplementary material for: Predicting Clinical Sensitivities of PDGFRA Exon 18 Mutations to Imatinib and Avapritinib to Optimize Gastrointestinal Stromal Tumor Treatment
Source: Cancer Res Commun. 2026 Jul 6;6(7):1573–91. doi: 10.1158/2767-9764.CRC-26-0093 (PMC13333789; doi:10.1158/2767-9764.CRC-26-0093)
Supplement: Supplementary Table S1 — Table S1. List of oligo sequences for each PDGFRA mutation modeled successfully in either Ba/F3s or CHOs. [file crc-26-0093_supplementary_table_s1_suppst1.pdf]

Supplementary Table 1

Oligo sequences for PDGFRA D842X point mutation library

Amino Acid (AA) #	834	835	836	837	838	839	840	841	**842**	843	844	845	846	847	848	849	850	851	852	853	854	855
**Wildtype AA Seq.**	I	C	D	F	G	L	A	R	**D**	I	M	H	D	S	N	Y	V	S	K	G	S	T
**Wildtype Nucleotide Seq.**	ATC	TGT	GAC	TTT	GGC	CTG	GCC	AGA	**GAT**	ATC	ATG	CAT	GAT	TCG	AAC	TAT	GTG	TCG	AAA	GGC	AGT	ACC
**D842A**	ATC	TGT	GAC	TTT	GGC	CTG	GCC	AGA	**GCG**	ATA	ATG	CAC	GAT	TCT	AAC	TAT	GTG	TCG	AAA	GGC	AGT	ACC
**D842C**	ATC	TGT	GAC	TTT	GGC	CTG	GCC	AGA	**TGC**	ATA	ATG	CAC	GAT	AGT	AAC	TAT	GTG	TCG	AAA	GGC	AGT	ACC
**D842D**	ATC	TGT	GAC	TTT	GGC	CTG	GCC	AGA	**GAC**	ATA	ATG	CAC	GAC	TCC	AAC	TAT	GTG	TCG	AAA	GGC	AGT	ACC
**D842E**	ATC	TGT	GAC	TTT	GGC	CTG	GCC	AGA	**GAA**	ATA	ATG	CAC	GAT	AGC	AAC	TAT	GTG	TCG	AAA	GGC	AGT	ACC
**D842F**	ATC	TGT	GAC	TTT	GGC	CTG	GCC	AGA	**TTC**	ATA	ATG	CAT	GAT	TCT	AAC	TAT	GTG	TCG	AAA	GGC	AGT	ACC
**D842G**	ATC	TGT	GAC	TTT	GGC	CTG	GCC	AGA	**GGA**	ATA	ATG	CAT	GAC	AGT	AAC	TAT	GTG	TCG	AAA	GGC	AGT	ACC
**D842H**	ATC	TGT	GAC	TTT	GGC	CTG	GCC	AGA	**CAC**	ATA	ATG	CAT	GAT	TCC	AAC	TAT	GTG	TCG	AAA	GGC	AGT	ACC
**D842I**	ATC	TGT	GAC	TTT	GGC	CTG	GCC	AGA	**ATT**	ATT	ATG	CAT	GAT	TCC	AAC	TAT	GTG	TCG	AAA	GGC	AGT	ACC
**D842K**	ATC	TGT	GAC	TTT	GGC	CTG	GCC	AGA	**AAG**	ATT	ATG	CAT	GAC	AGT	AAC	TAT	GTG	TCG	AAA	GGC	AGT	ACC
**D842L**	ATC	TGT	GAC	TTT	GGC	CTG	GCC	AGA	**CTG**	ATT	ATG	CAT	GAC	TCT	AAC	TAT	GTG	TCG	AAA	GGC	AGT	ACC
**D842M**	ATC	TGT	GAC	TTT	GGC	CTG	GCC	AGA	**ATG**	ATT	ATG	CAT	GAC	AGC	AAC	TAT	GTG	TCG	AAA	GGC	AGT	ACC
**D842N**	ATC	TGT	GAC	TTT	GGC	CTG	GCC	AGA	**AAC**	ATT	ATG	CAC	GAT	AGC	AAC	TAT	GTG	TCG	AAA	GGC	AGT	ACC
**D842P**	ATC	TGT	GAC	TTT	GGC	CTG	GCC	AGA	**CCA**	ATT	ATG	CAC	GAC	TCT	AAC	TAT	GTG	TCG	AAA	GGC	AGT	ACC
**D842Q**	ATC	TGT	GAC	TTT	GGC	CTG	GCC	AGA	**CAA**	ATT	ATG	CAC	GAC	AGT	AAC	TAT	GTG	TCG	AAA	GGC	AGT	ACC
**D842R**	ATC	TGT	GAC	TTT	GGC	CTG	GCC	AGA	**CGT**	ATC	ATG	CAC	GAT	TCC	AAC	TAT	GTG	TCG	AAA	GGC	AGT	ACC
**D842S**	ATC	TGT	GAC	TTT	GGC	CTG	GCC	AGA	**TCT**	ATC	ATG	CAC	GAT	AGC	AAC	TAT	GTG	TCG	AAA	GGC	AGT	ACC
**D842T**	ATC	TGT	GAC	TTT	GGC	CTG	GCC	AGA	**ACT**	ATC	ATG	CAC	GAT	TCT	AAC	TAT	GTG	TCG	AAA	GGC	AGT	ACC
**D842V**	ATC	TGT	GAC	TTT	GGC	CTG	GCC	AGA	**GTA**	ATC	ATG	CAC	GAT	AGT	AAC	TAT	GTG	TCG	AAA	GGC	AGT	ACC
**D842W**	ATC	TGT	GAC	TTT	GGC	CTG	GCC	AGA	**TGG**	ATC	ATG	CAT	GAC	TCC	AAC	TAT	GTG	TCG	AAA	GGC	AGT	ACC
**D842* (STOP)**	ATC	TGT	GAC	TTT	GGC	CTG	GCC	AGA	**TGA**	ATC	ATG	CAT	GAC	AGC	AAC	TAT	GTG	TCG	AAA	GGC	AGT	ACC
**D842Y**	ATC	TGT	GAC	TTT	GGC	CTG	GCC	AGA	**TAC**	ATC	ATG	CAT	GAC	TCT	AAC	TAT	GTG	TCG	AAA	GGC	AGT	ACC

Oligo sequences for PDGFRA D842_D846delinsX mutation library

Amino Acid (AA) #	834	835	836	837	838	839	840	841	**842**	843	844	845	846	847	848	849	850	851	852	853	854	855
**Wildtype AA Seq.**	I	C	D	F	G	L	A	R	**D**	I	M	H	D	S	N	Y	V	S	K	G	S	T
**Wildtype Nucleotide Seq.**	ATC	TGT	GAC	TTT	GGC	CTG	GCC	AGA	**GAT**	ATC	ATG	CAT	GAT	TCG	AAC	TAT	GTG	TCG	AAA	GGC	AGT	ACC
**D842_D846delinsA**	ATC	TGT	GAC	TTT	GGC	CTG	GCC	AGA	**GCG**	-	-	-	-	TCT	AAC	TAT	GTG	TCG	AAA	GGC	AGT	ACC
**D842_D846delinsC**	ATC	TGT	GAC	TTT	GGC	CTG	GCC	AGA	**TGT**	-	-	-	-	TCT	AAC	TAT	GTG	TCG	AAA	GGC	AGT	ACC
**D842_D846delinsD**	ATC	TGT	GAC	TTT	GGC	CTG	GCC	AGA	**GAT**	-	-	-	-	TCA	AAC	TAT	GTG	TCG	AAA	GGC	AGT	ACC
**D842_D846delinsE**	ATC	TGT	GAC	TTT	GGC	CTG	GCC	AGA	**GAG**	-	-	-	-	TCT	AAC	TAT	GTG	TCG	AAA	GGC	AGT	ACC
**D842_D846delinsF**	ATC	TGT	GAC	TTT	GGC	CTG	GCC	AGA	**TTT**	-	-	-	-	TCC	AAC	TAT	GTG	TCG	AAA	GGC	AGT	ACC
**D842_D846delinsG**	ATC	TGT	GAC	TTT	GGC	CTG	GCC	AGA	**GGT**	-	-	-	-	TCC	AAC	TAT	GTG	TCG	AAA	GGC	AGT	ACC
**D842_D846delinsH**	ATC	TGT	GAC	TTT	GGC	CTG	GCC	AGA	**CAT**	-	-	-	-	TCA	AAC	TAT	GTG	TCG	AAA	GGC	AGT	ACC
**D842_D846delinsI**	ATC	TGT	GAC	TTT	GGC	CTG	GCC	AGA	**ATA**	-	-	-	-	TCC	AAC	TAT	GTG	TCG	AAA	GGC	AGT	ACC
**D842_D846delinsK**	ATC	TGT	GAC	TTT	GGC	CTG	GCC	AGA	**AAG**	-	-	-	-	TCT	AAC	TAT	GTG	TCG	AAA	GGC	AGT	ACC
**D842_D846delinsL**	ATC	TGT	GAC	TTT	GGC	CTG	GCC	AGA	**CTG**	-	-	-	-	TCT	AAC	TAT	GTG	TCG	AAA	GGC	AGT	ACC
**D842_D846delinsM**	ATC	TGT	GAC	TTT	GGC	CTG	GCC	AGA	**ATG**	-	-	-	-	TCA	AAC	TAT	GTG	TCG	AAA	GGC	AGT	ACC
**D842_D846delinsN**	ATC	TGT	GAC	TTT	GGC	CTG	GCC	AGA	**AAT**	-	-	-	-	TCT	AAC	TAT	GTG	TCG	AAA	GGC	AGT	ACC
**D842_D846delinsP**	ATC	TGT	GAC	TTT	GGC	CTG	GCC	AGA	**CCG**	-	-	-	-	TCC	AAC	TAT	GTG	TCG	AAA	GGC	AGT	ACC
**D842_D846delinsQ**	ATC	TGT	GAC	TTT	GGC	CTG	GCC	AGA	**CAG**	-	-	-	-	TCA	AAC	TAT	GTG	TCG	AAA	GGC	AGT	ACC
**D842_D846delinsR**	ATC	TGT	GAC	TTT	GGC	CTG	GCC	AGA	**CGT**	-	-	-	-	TCT	AAC	TAT	GTG	TCG	AAA	GGC	AGT	ACC
**D842_D846delinsS**	ATC	TGT	GAC	TTT	GGC	CTG	GCC	AGA	**AGT**	-	-	-	-	TCA	AAC	TAT	GTG	TCG	AAA	GGC	AGT	ACC
**D842_D846delinsT**	ATC	TGT	GAC	TTT	GGC	CTG	GCC	AGA	**ACG**	-	-	-	-	TCA	AAC	TAT	GTG	TCG	AAA	GGC	AGT	ACC

**D842_D846delinsV**	ATC	TGT	GAC	TTT	GGC	CTG	GCC	AGA	**GTG**	-	-	-	-	TCT	AAC	TAT	GTG	TCG	AAA	GGC	AGT	ACC
**D842_D846delinsW**	ATC	TGT	GAC	TTT	GGC	CTG	GCC	AGA	**TGG**	-	-	-	-	TCA	AAC	TAT	GTG	TCG	AAA	GGC	AGT	ACC
**D842_D846delins* (STOP)**	ATC	TGT	GAC	TTT	GGC	CTG	GCC	AGA	**TGA**	-	-	-	-	TCA	AAC	TAT	GTG	TCG	AAA	GGC	AGT	ACC
**D842_D846delinsY**	ATC	TGT	GAC	TTT	GGC	CTG	GCC	AGA	**TAT**	-	-	-	-	TCA	AAC	TAT	GTG	TCG	AAA	GGC	AGT	ACC

Oligo sequences for additional PDGFRA 842-position mutations

**Amino Acid (AA) #**	834	835	836	837	838	839	840	841	**842**	843	844	845	846	847	848	849	850	851	852	853	854	855
**Wildtype AA Seq.**	I	C	D	F	G	L	A	R	**D**	I	M	H	D	S	N	Y	V	S	K	G	S	T
**Wildtype Nucleotide Seq.**	ATC	TGT	GAC	TTT	GGC	CTG	GCC	AGA	**GAT**	ATC	ATG	CAT	GAT	TCG	AAC	TAT	GTG	TCG	AAA	GGC	AGT	ACC
**D842_I843delinsV**	ATC	TGT	GAC	TTT	GGC	CTG	GCC	AGA	**GTC**	-	ATG	CAT	GAT	TCG	AAC	TAT	GTG	TCG	AAA	GGC	AGT	ACC
**D842_M844delinsV**	ATC	TGT	GAC	TTT	GGC	CTG	GCC	AGA	**GTG**	-	-	CAC	GAC	TCG	AAC	TAT	GTG	TCG	AAA	GGC	AGT	ACC
**D842_H845delinsV**	ATC	TGT	GAC	TTT	GGC	CTG	GCC	AGA	**GTG**	-	-	-	GAC	TCG	AAC	TAT	GTG	TCG	AAA	GGC	AGT	ACC
**D842del**	ATC	TGT	GAC	TTT	GGC	CTG	GCC	AGA	**-**	ATC	ATG	CAT	GAT	TCG	AAC	TAT	GTG	TCG	AAA	GGC	AGT	ACC
**D842_I843del**	ATC	TGT	GAC	TTT	GGC	CTG	GCC	AGA	**-**	**-**	ATG	CAT	GAT	TCG	AAC	TAT	GTG	TCG	AAA	GGC	AGT	ACC
**D842_M844del**	ATC	TGT	GAC	TTT	GGC	CTG	GCC	AGA	**-**	-	-	CAT	GAT	TCG	AAC	TAT	GTG	TCG	AAA	GGC	AGT	ACC
**D842_S847delinsAT**	ATC	TGT	GAC	TTT	GGC	CTG	GCC	AGA	**GCC**	-	-	-	-	**ACG**	AAC	TAT	GTG	TCG	AAA	GGC	AGT	ACC

Supplementary Table 1: List of oligo sequences for each PDGFRA mutation modeled successfully in either Ba/F3s or CHOs. Purple shaded cells indicate the 842-position codon mutation's nucleotide sequence, red shaded cells indicate an additional mutation, dashes represent a deleted residue, and green shaded cells indicate a silent mutation introduced as a sequencing barcode.
