# Supplementary material for: Predicting Clinical Sensitivities of PDGFRA Exon 18 Mutations to Imatinib and Avapritinib to Optimize Gastrointestinal Stromal Tumor Treatment
Source: Cancer Res Commun. 2026 Jul 6;6(7):1573–91. doi: 10.1158/2767-9764.CRC-26-0093 (PMC13333789; doi:10.1158/2767-9764.CRC-26-0093)
Supplement: Supplementary Table S2 — Table S2. List of primer sequences used to PCR amplify and sequence PDGFRA cDNA. [file crc-26-0093_supplementary_table_s2_suppst2.pdf]

Supplementary Table 2

Primers 5' to 3'	PDGFRA cDNA region amplified
GCTCGTTTAGTGAACCGTCAG	Exons 1-4
CCCCTCACTGTTGTGTAAGG	Exons 1-4
GGGACTTTGATTTCTTCCAG	Exons 4-6
GGCCCTATATCTGTGAGGC	Exons 5-7
CCACCTCCCAGGATATCCTGGCTG	Exons 7-10
GTTCTGAACTCACGGTGGCT	Exons 11-13
CTTCAACCACCTTCCCAAAC	Exons 11-13
CCGATGCAGCTGCCTTATG	Exons 12-14
GTAAGGCCTTCTGAGTTATC	Exons 15-17
CCGACATCCAGAGATCACTCTATG	Exons 16-18
TATCAAGTTGCCCGAGGAAT	Exons 18-20
GATCTCCCAGAGCAGAATGCC	Exons 18-20
GTGAGCCGGAGAAGAGACCC	Exons 21-23
CACACCGGCCTTATTCCAAG	Exons 21-23

Supplementary Table 2: List of primer sequences used to PCR amplify and sequence *PDGFRA* cDNA.
