# Supplementary material for: Predicting Clinical Sensitivities of PDGFRA Exon 18 Mutations to Imatinib and Avapritinib to Optimize Gastrointestinal Stromal Tumor Treatment
Source: Cancer Res Commun. 2026 Jul 6;6(7):1573–91. doi: 10.1158/2767-9764.CRC-26-0093 (PMC13333789; doi:10.1158/2767-9764.CRC-26-0093)
Supplement: Supplementary Table S3 — Table S3. List of primary mutations seen in our 1379 PDGFRA-mutant GIST case cohort. Mutations are separated out by exon region in which they are found. [file crc-26-0093_supplementary_table_s3_suppst3.pdf]

Supplementary Table 3

Database includes data from European Sarcoma centers, AACR GENIE v18.0, Oregon Health and Science University (OHSU), and Foundation Medicine

Exon 12 Cases (n = 170)

Cases from each source:
European Sarcoma Centers n = 48
AACR GENIE n = 34
OHSU n = 48
Foundation Medicine n = 40

Mutation	Count
V561D	89
S566_E571delinsR	11
W559_R560del	6
I565_S566delinsEIRWRVIESI	5
E556_I565dup	4
R585_E587del	3
S566_571EdelinsR	3
S566_E571delinsK	3
V594_L595delinsREFPRDGLV	2
X552_splice	2
Y555C	2
D576N	1
D583_W586del	1
E556_D576dup	1
E558_I585dup	1
E563Vfs*8	1
H570R	1
I557I	1
I562_E571delinsK	1
I565Nfs*7	1
K550_Q556delinsI	1
L576P	1
L580P	1
L593I	1
M578_S584del	1
P553Lfs*39	1
P581_W586delinsRC	1
P581delinsMMQLS	1
P589_R590delinsPYDSRWEFP	1
R558_I565del	1
R558C	1
R558H	1
R560_V561del	1
R560_V651delinsC	1
S556_E571delinsR	1
S566_E571delinsQ	1
S566R & P567_E571del	1
V559D	1
V561_P567del	1
W559_H569delIM570I	1
W559_R560del & N659K	1
W586_E587delinsYDSRW	1
Y555C & N659D	1
Y574H	1
Y582_R585del	1
L595_G596delinsEMEFPRDGLVL	1
I562_E563delinsEIRWRVI	1
I562_E563delinsVIRWRVI	1
P589_R590delinsTRWEFP	1
V594_L595delinsPYDSRWEFPRDGLV	1
Y582_D583delinsYDSRW	1

Exon 14 Cases (n = 87)

Cases from each source:
European Sarcoma Centers n = 24
AACR GENIE n = 13
OHSU n = 32
Foundation Medicine n = 18

Mutation	Count
N659K	57
N659Y	26
G652E	1
K646E	1
L651_G652insTHL	1
N659S	1

Exon 18 Cases (n = 1122)

Cases from each source:
European Sarcoma Centers n = 308
AACR GENIE n = 153
OHSU n = 393
Foundation Medicine n = 268

Mutation	Count
D842V	747
I843_D846del	186
D842Y	19
D842_D846delinsE	15
I843_S847delinsT	15
D842_M844del	14
D842_D846delinsA	12
D846Y	10
D842I	8
D842_H845del	7
D842_D846delinsN	5
M844_S847del	5
Y849C	5
R841_D842del	3
Y849D	3
D842_D846delinsT	2
D842_I843del	2
D842_S847delinsCL	2
D842_S847delinsEI	2
H845_N848delinsP	2
I843_S847delinsA	2
I843_S847delinsM	2
A821T	1
A827T	1
D842_D846del	1
D842_D846delinsAY	1
D842_D846delinsH	1
D842_D846delinsL	1
D842_D846delinsR	1
D842_D846delinsS	1
D842_H845delinsV	1
D842_I843delinsV	1
D842_N848delinsVDV	1
D842_N848delinsVRDV	1
D842_S847delinsA	1
D842_S847delinsAM	1
D842_S847delinsANL	1
D842_S847delinsAT	1
D842_S847delinsE	1
D842_S847delinsEG	1
D842_S847delinsEL	1
D842_S847delinsESL	1
D842_S847delinsGT	1
D842_S847delinsVL	1
D842_S847delinsVMP	1
D842A	1
D842del	1
D842F	1
D842G	1
D842H	1
D842H & D846Y	1
D842I & I843M	1
D842L	1
D842Tfs*18	1
D842V & K833N	1
D842V & T1052M	1
D842Y & D846H	1
D846del	1
D846N	1
H845_D846delinsPN	1
H845P & D846H	1
I831L	1
I831T	1
I843_D846delinsH	1
I843_D846delinsN	1
I843_S847delinsL	1
K833R	1
M844_S847delinsP	1
R841_I843delinsKV	1
R841_N848delinsVDV	1
R841_H845delinsKLCV	1
R841K & D842V	1
R841S	1
S847_Y849del	1
S847L	1
V824I	1

Supplementary Table 3: List of primary mutations seen in our 1379 PDGFRA-mutant GIST case cohort. Mutations are separated out by exon region in which they are found. Number of cases from each source also listed.
