# Supplementary material for: Predicting Clinical Sensitivities of PDGFRA Exon 18 Mutations to Imatinib and Avapritinib to Optimize Gastrointestinal Stromal Tumor Treatment
Source: Cancer Res Commun. 2026 Jul 6;6(7):1573–91. doi: 10.1158/2767-9764.CRC-26-0093 (PMC13333789; doi:10.1158/2767-9764.CRC-26-0093)
Supplement: Supplementary Table S4 — Table S4. Imatinib IC50 values and 95% confidence intervals (CIs) of PDGFRA mutations modeled in Ba/F3 and CHO cells. [file crc-26-0093_supplementary_table_s4_suppts4.pdf]

Supplementary Table 4

Ba/F3 cell lines				
**Mutation**	**# of experiments**	**Calculated IC_{50} [nM]**	**95% CI- lower [nM]**	**95% CI- upper [nM]**
D842V	7	574.6	471.5	710.9
D842I	4	581.1	385.6	849.4
D842L	5	520.6	402.9	684.8
D842M	4	402.2	334.6	485.6
D842F	5	333.8	233.6	494
D842Y	6	341.7	283	411.1
D842Q	3	30.39	25.2	36.38
D842R	3	108.2	72.15	176.4
D842_D846delinsA	4	33.95	24.66	45.25
D842_D846delinsV	5	264.1	219.6	317.4
D842_D846delinsI	3	1848	1288	3178
D842_D846delinsL	4	823.5	546.5	1639
D842_D846delinsM	3	462.7	356.7	597
D842_D846delinsF	6	1077	2261	696.9
D842_D846delinsY	4	261.7	200.5	341
D842_D846delinsW	4	185.7	158.1	218.5
D842_D846delinsS	4	17.02	10.91	23.66
D842_D846delinsT	3	88.39	71.86	108.3
D842_D846delinsN	4	31.28	21.4	43.55
D842_D846delinsQ	3	54.16	43.74	67.99
D842_D846delinsC	3	41.16	30.18	54.56
D842_D846delinsG	4	17.15	12.75	21.67
D842_D846delinsP	4	57.01	41.73	74.96
D842_D846delinsR	3	44.6	32.53	59.35
D842_D846delinsH	4	91.87	58.48	139.6
D842_D846delinsK	4	28.19	23.44	33.41
D842_D846delinsE	4	25.77	21.93	29.91
D842_D846delinsD	4	6.86	3.799	10.23
D842_I843delinsV	4	266.5	210.8	335.3
D842_H845delinsV	3	223.2	158.2	313.2
D842_S847delinsAT	3	16.81	13.17	20.79
D842del	4	1117	913.4	1386
D842_M844del	5	19.4	15.02	24.44

CHO cell lines				
**Mutation**	**# of experiments**	**Calculated IC_{50} [nM]**	**95% CI- lower [nM]**	**95% CI- upper [nM]**
D842A	4	47.74	35.34	62.37
D842V	5	582.4	354	1211
D842I	3	748.1	446.3	1713
D842L	5	337.4	214.4	544
D842M	6	641.3	412.2	1178
D842F	5	750.4	493.4	1442
D842Y	3	274	193.2	386.5
D842W	3	553.3	425	743.1
D842S	3	90.04	55.2	138.7
D842T	4	47.7	25.88	78.53
D842N	3	76.15	49.59	114.5
D842Q	3	22.76	7.114	45.54
D842C	4	78.15	47.88	121.2
D842G	3	35.36	20.31	56.2
D842P	3	56	42.08	73.94
D842R	4	73.92	55.06	99.18
D842H	6	93.79	58.06	141.6
D842K	6	61.49	41.95	89.73
D842E	3	11.4	8.046	15.02
I843_D846del	6	11.6	9.032	14.3

Supplementary Table 4: Imatinib IC_{50} values and 95% confidence intervals (CIs) of PDGFRA mutations modeled in Ba/F3 and CHO cells. The number of independent immunoblotting experiments used to calculate the IC_{50}, along with the calculated IC_{50} and 95% CI (lower and upper IC_{50} in nM), are listed.
