# Supplementary material for: Predicting Clinical Sensitivities of PDGFRA Exon 18 Mutations to Imatinib and Avapritinib to Optimize Gastrointestinal Stromal Tumor Treatment
Source: Cancer Res Commun. 2026 Jul 6;6(7):1573–91. doi: 10.1158/2767-9764.CRC-26-0093 (PMC13333789; doi:10.1158/2767-9764.CRC-26-0093)
Supplement: Supplementary Table S5 — Table S5. Avapritinib IC50 values and 95% confidence intervals (CIs) of PDGFRA mutations modeled in Ba/F3 and CHO cells. [file crc-26-0093_supplementary_table_s5_suppst5.pdf]

Supplementary Table 5

Ba/F3 cell lines				
**Mutation**	**# of experiments**	**Calculated IC_{50} [nM]**	**95% CI- lower [nM]**	**95% CI- upper [nM]**
D842V	3	3.521	2.668	4.514
D842I	3	4.553	3.349	6.063
D842L	3	11.23	8.843	14.21
D842M	5	9.147	6.101	13.58
D842F	4	8.799	6.735	11.44
D842Y	4	16.65	13.11	21.48
D842R	3	4.508	3.335	5.898
D842_D846delinsA	4	2.902	1.764	4.464
D842_D846delinsV	4	1.478	1.27	1.703
D842_D846delinsL	4	2.018	1.612	2.486
D842_D846delinsF	4	5.763	4.551	6.469
D842_D846delinsY	4	6.627	4.295	9.659
D842_D846delinsW	3	7.766	5.376	11.19
D842_D846delinsS	4	4.237	2.903	5.904
D842_D846delinsT	3	2.484	2.059	2.984
D842_D846delinsN	3	2.706	2.245	3.236
D842_D846delinsQ	3	2.823	2.259	3.487
D842_D846delinsC	3	1.429	1.134	1.747
D842_D846delinsG	3	3.813	3.14	4.564
D842_D846delinsP	4	1.491	1.037	1.998
D842_D846delinsH	3	1.801	1.451	2.187
D842_D846delinsK	3	1.826	1.255	2.538
D842_D846delinsE	4	1.688	1.258	2.177
D842_D846delinsD	3	6.41	5.796	7.113
D842_S847delinsAT	3	2.621	2.215	3.091
D842_I843delinsV	3	2.411	1.489	3.55
D842_H845delinsV	3	3.956	3.148	4.872
D842_M844del	4	9.005	6.188	11.94

CHO cell lines				
**Mutation**	**# of experiments**	**Calculated IC_{50} [nM]**	**95% CI- lower [nM]**	**95% CI- upper [nM]**
D842A	3	4.366	3.322	5.595
D842V	3	5.19	3.903	6.692
D842I	4	3.419	1.539	5.93
D842L	3	4.465	3.287	5.894
D842M	3	2.466	1.326	3.864
D842F	3	12.29	9.018	16.68
D842Y	6	2.998	1.508	4.705
D842W	3	10.86	8.047	14.52
D842S	4	4.679	3.028	6.654
D842T	3	10.55	7.781	14.19
D842N	4	11.57	8.056	15.83
D842Q	3	9.735	6.909	13.61
D842C	4	6.215	4.446	8.29
D842G	4	8.975	5.696	13.2
D842P	4	6.422	3.981	9.227
D842R	3	8.466	5.829	12.04
D842H	3	6.321	4.092	9.182
D842K	3	6.841	4.102	11.5
D842E	3	5.956	0.898	17.15
I843_D846del	4	3.88	2.526	5.596
D842V + V658A	4	80.05	59.72	110.7

Supplementary Table 5: Avapritinib IC_{50} values and 95% confidence intervals (CIs) of PDGFRA mutations modeled in Ba/F3 and CHO cells. The number of independent immunoblotting experiments used to calculate the IC_{50}, along with the calculated IC_{50} and 95% CI (lower and upper IC_{50} in nM), are listed.
