# Supplementary material for: Predicting Clinical Sensitivities of PDGFRA Exon 18 Mutations to Imatinib and Avapritinib to Optimize Gastrointestinal Stromal Tumor Treatment
Source: Cancer Res Commun. 2026 Jul 6;6(7):1573–91. doi: 10.1158/2767-9764.CRC-26-0093 (PMC13333789; doi:10.1158/2767-9764.CRC-26-0093)
Supplement: Supplementary Table S6 — Table S6. List of PDGFRA mutations that were observed in the cases within our cohort, along with information about whether these mutations were modeled in vitro. [file crc-26-0093_supplementary_table_s6_suppst6.pdf]

Supplementary Table 6

D842X mutations observed in 1122 case cohort						
842 AA Class	Mutation	842 AA	Modeled	Cases observed	Biochemical or Predicted Sensitivity	Sensitivity Classification
Hydrophobic	D842A	A	Yes	1	Biochemical	Sensitive
	D842V	V	Yes	747	Biochemical	Resistant
	D842I	I	Yes	8	Biochemical	Resistant
	D842L	L	Yes	1	Biochemical	Resistant
	D842M	M	Yes	0	Biochemical	Resistant
	D842F	F	Yes	1	Biochemical	Resistant
	D842Y	Y	Yes	19	Biochemical	Resistant
	D842W	W	Yes	0	Biochemical	Resistant
Polar Uncharged	D842S	S	Yes	0	Biochemical	Intermediate Resistance
	D842T	T	Yes	0	Biochemical	Intermediate Resistance
	D842N	N	Yes	0	Biochemical	Intermediate Resistance
	D842Q	Q	Yes	0	Biochemical	Sensitive
Special Case	D842C	C	Yes	0	Biochemical	Intermediate Resistance
	D842G	G	Yes	1	Biochemical	Sensitive
	D842P	P	Yes	0	Biochemical	Sensitive
Positively Charged	D842R	R	Yes	0	Biochemical	Intermediate Resistance (CHO), Sensitive (Ba/F3)
	D842H	H	Yes	1	Biochemical	Intermediate Resistance
	D842K	K	Yes	0	Biochemical	Intermediate Resistance
Negatively Charged	D842E	E	Yes	0	Biochemical	Sensitive

D842_D846delinsX mutations observed in 1122 case cohort						
842 AA Class	Mutation	842 AA	Modeled	Cases observed	Biochemical or Predicted Sensitivity	Sensitivity Classification
Hydrophobic	D842_D846delinsA	A	Yes	12	Biochemical	Sensitive
	D842_D846delinsV	V	Yes	0	Biochemical	Resistant
	D842_D846delinsI	I	Yes	0	Biochemical	Resistant
	D842_D846delinsL	L	Yes	1	Biochemical	Resistant
	D842_D846delinsM	M	Yes	0	Biochemical	Resistant
	D842_D846delinsF	F	Yes	0	Biochemical	Resistant
	D842_D846delinsY	Y	Yes	0	Biochemical	Resistant
	D842_D846delinsW	W	Yes	0	Biochemical	Intermediate Resistance
Polar Uncharged	D842_D846delinsS	S	Yes	1	Biochemical	Sensitive
	D842_D846delinsT	T	Yes	2	Biochemical	Sensitive
	D842_D846delinsN	N	Yes	5	Biochemical	Sensitive
	D842_D846delinsQ	Q	Yes	0	Biochemical	Sensitive
Special Case	D842_D846delinsC	C	Yes	0	Biochemical	Sensitive
	D842_D846delinsG	G	Yes	0	Biochemical	Sensitive
	D842_D846delinsP	P	Yes	0	Biochemical	Sensitive
Positively Charged	D842_D846delinsR	R	Yes	1	Biochemical	Sensitive
	D842_D846delinsH	H	Yes	1	Biochemical	Sensitive
	D842_D846delinsK	K	Yes	0	Biochemical	Sensitive
Negatively Charged	D842_D846delinsE	E	Yes	15	Biochemical	Sensitive
	D842_D846delinsD*	D	Yes	193	Biochemical	Sensitive

Other point mutations observed in 1122 case cohort						
842 AA Class	Mutation	842 AA	Modeled	Cases observed	Biochemical or Predicted Sensitivity	Sensitivity Classification
Hydrophobic	D842V & K833N	V	No	1	Predicted	Resistant
	D842V & T1052M	V	No	1	Predicted	Resistant
	R841K & D842V	V	No	1	Predicted	Resistant
	D842Y & D846H	Y	No	1	Predicted	Resistant
	D842I & I843M	I	No	1	Predicted	Resistant
Polar Uncharged	D842Tfs*18	T	No	1	Predicted	Sensitive
Positively Charged	D842H & D846Y	H	No	1	Predicted	Intermediate Resistance
Negatively Charged	A821T	D	No	1	Predicted	Sensitive
	V824I	D	No	1	Predicted	Resistant
	A827T	D	No	1	Predicted	Sensitive
	I831L	D	No	1	Predicted	Sensitive
	I831T	D	No	1	Predicted	Sensitive
	K833R	D	No	1	Predicted	Sensitive
	R841S	D	No	1	Predicted	Sensitive
	H845P & D846H	D	No	1	Predicted	Sensitive
	D846Y	D	No	10	Predicted	Sensitive
	D846N	D	No	1	Predicted	Sensitive
	S847L	D	No	1	Predicted	Sensitive
	Y849D	D	No	3	Predicted	Sensitive
	Y849C	D	No	5	Predicted	Sensitive

Other in/del mutations observed in 1122 case cohort						
842 AA Class	Mutation	842 AA	Modeled	Cases observed	Biochemical or Predicted Sensitivity	Sensitivity Classification
Hydrophobic	D842_S847delinsA	A	No	1	Predicted	Sensitive
	D842_S847delinsAM	A	No	1	Predicted	Sensitive
	D842_S847delinsAT	A	Yes	1	Biochemical	Sensitive
	D842_S847delinsANL	A	No	1	Predicted	Sensitive
	D842_D846delinsAY	A	No	1	Predicted	Sensitive
	D842_S847delinsVL	V	No	1	Predicted	Resistant
	D842_S847delinsVMP	V	No	1	Predicted	Resistant
	D842_N848delinsVDV	V	No	1	Predicted	Resistant
	D842_N848delinsVRDV	V	No	1	Predicted	Resistant
	D842_H845delinsV	V	Yes	1	Biochemical	Intermediate Resistance
	D842_I843delinsV	V	Yes	1	Biochemical	Resistant
	R841_D842del	M	No	3	Predicted	Resistant
	D842_I843del	M	No	2	Predicted	Resistant
	D842del	I	Yes	1	Biochemical	Resistant
	R841_H845delinsKLCV	L	No	1	Predicted	Resistant

	R841_I843delinsKV	V	No	1	Predicted	Resistant
Polar Uncharged	D842_D846del	S	No	1	Predicted	Sensitive
Special Case	D842_S847delinsCL	C	No	2	Predicted	Sensitive
	D842_S847delinsGT	G	No	1	Predicted	Sensitive
Positively Charged	D842_M844del	H	Yes	14	Biochemical	Sensitive
Negatively Charged	R841_N848delinsVDV	D	No	1	Predicted	Sensitive
	I843_S847delinsA	D	No	2	Predicted	Sensitive
	I843_S847delinsT	D	No	15	Predicted	Sensitive
	I843_D846delinsH	D	No	1	Predicted	Sensitive
	I843_D846delinsN	D	No	1	Predicted	Sensitive
	I843_S847delinsL	D	No	1	Predicted	Sensitive
	I843_S847delinsM	D	No	2	Predicted	Sensitive
	D842_S847delinsE	E	No	1	Predicted	Sensitive
	D842_S847delinsEG	E	No	1	Predicted	Sensitive
	D842_S847delinsEL	E	No	1	Predicted	Sensitive
	D842_S847delinsESL	E	No	1	Predicted	Sensitive
	D842_S847delinsEI	E	No	2	Predicted	Sensitive
	M844_S847delinsP	D	No	1	Predicted	Sensitive
	M844_S847del	D	No	5	Predicted	Sensitive
	H845_D846delinsPN	D	No	1	Predicted	Sensitive
	H845_N848delinsP	D	No	2	Predicted	Sensitive
	D846del	D	No	1	Predicted	Sensitive
	S847_Y849del	D	No	1	Predicted	Sensitive

Supplementary Table 6: List of PDGFRA mutations that were observed in the cases within our cohort, along with information about whether these mutations were modeled *in vitro* . Mutation, 842-position amino acid, case counts, and imatinib sensitivity predictions are listed. For mutations modeled *in vitro* , imatinib sensitivity is classified based on biochemical IC_{50}. Those mutations not modeled have predicted imatinib sensitivity classifications solely based on biochemical data of other mutations with the same 842-position amino acid. *includes cases of I843_D846del and D842_H845del, as these mutations are synonymous to D842_D846delinsD at the protein level.
