# Supplementary material for: Predicting Clinical Sensitivities of PDGFRA Exon 18 Mutations to Imatinib and Avapritinib to Optimize Gastrointestinal Stromal Tumor Treatment
Source: Cancer Res Commun. 2026 Jul 6;6(7):1573–91. doi: 10.1158/2767-9764.CRC-26-0093 (PMC13333789; doi:10.1158/2767-9764.CRC-26-0093)
Supplement: Supplementary Table S7 — Table S7. Clinical responses of advanced PDGFRA-mutant GIST patients treated with first-line imatinib. [file crc-26-0093_supplementary_table_s7_suppst7.pdf]

Supplementary Table 7

Patient ID	Source: PubMed ID (if applicable)	Mutation	PFS in Months	D842V	Exon 18-Sens	Exon 18-Res	Combined Resistant (D842V + Exon 18-Res)
1	Life Raft Group	D842V	17.7	1			1
2	Life Raft Group	D842V	31.08	1			1
3	Life Raft Group	D842V	7.95	1			1
4	Life Raft Group	D842V	7.42	1			1
5	Life Raft Group	D842V	5.62	1			1
6	Life Raft Group	D842V	3.02	1			1
7	Life Raft Group	D842V	2.98	1			1
8	Life Raft Group	D842V	1.87	1			1
9	Life Raft Group	D842V	2.23	1			1
10	Life Raft Group	D842V	1.35	1			1
11	Life Raft Group	D842V	1.44	0			0
12	Life Raft Group	D842V	3.77	1			1
13	Life Raft Group	D842V	1.05	0			0
14	Life Raft Group	D842_D846delinsE	79.77		1		
15	Life Raft Group	D846del	55.06		0		
16	Life Raft Group	G838D	42.97		1		
17	Life Raft Group	M844_S847delinsHDS	27.6		1		
18	Life Raft Group	Y849S	14.98		1		
19	Life Raft Group	D842_H845delinsV	0.95			0	0
20	EORTC: 22718859	D842_S847delinsVLIN	12			1	1
21	EORTC: 22718859	I843_D846del	35		0		
22	EORTC: 22718859	D842_S847delinsVL	66			0	0
23	EORTC: 22718859	I843_S847delinsT	8		0		
24	EORTC: 22718859	I843_D846del	12		1		
25	EORTC: 22718859	D842_H845del	3		0		
26	EORTC: 22718859	I843_S847del	12		0		
27	EORTC: 22718859	D846Y	55		1		
28	EORTC: 22718859	D842_M844del	30		1		
29	EORTC: 22718859	D842del	2			1	1
30	EORTC: 22718859	I843_H845del	30		1		
31	EORTC: 22718859	D846V	2		1		
32	EORTC: 22718859	D842_M844del	4		1		
33	EORTC: 22718859	D842_H845del	42		1		
34	EORTC: 22718859	D842_H845del	24		1		
35	EORTC: 22718859	I843_D846del	27		1		
36	EORTC: 22718859	D842_H845del	58		0		
37	EORTC: 22718859	D842V	1	1			1
38	EORTC: 22718859	D842V	6	1			1
39	EORTC: 22718859	D842V	61	0			0
40	EORTC: 22718859	D842V	4	1			1
41	EORTC: 22718859	D842V	35	0			0
42	EORTC: 22718859	D842V	1	1			1
43	EORTC: 22718859	D842V	2	1			1
44	EORTC: 22718859	D842V	2	1			1
45	EORTC: 22718859	D842V	16	0			0
46	EORTC: 22718859	D842V	2	1			1
47	EORTC: 22718859	D842V	2	1			1
48	EORTC: 22718859	D842V	6	1			1
49	EORTC: 22718859	D842V	0.1	0			0
50	EORTC: 22718859	D842V	5	1			1
51	EORTC: 22718859	D842V	13	1			1
52	EORTC: 22718859	D842V	41	1			1
53	EORTC: 22718859	D842V	3	1			1
54	EORTC: 22718859	D842V	5	1			1
55	EORTC: 22718859	D842V	2	1			1
56	EORTC: 22718859	D842V	15	1			1
57	EORTC: 22718859	D842V	11	1			1
58	EORTC: 22718859	D842V	2	1			1
59	EORTC: 22718859	D842V	8	1			1
60	EORTC: 22718859	D842V	3	1			1
61	EORTC: 22718859	D842V	2	1			1
62	EORTC: 22718859	D842V	2	1			1
63	EORTC: 22718859	D842V	3	1			1
64	EORTC: 22718859	D842V	3	1			1
65	EORTC: 22718859	D842V	3	1			1
66	EORTC: 22718859	D842V	1	1			1
67	EORTC: 22718859	D842V	3	1			1
68	EORTC: 22718859	D842V	6	1			1
69	Korean: 26130666	D842V	4.2	1			1
70	Korean: 26130666	D842V	2.7	0			0
71	Korean: 26130666	D842V	1.4	0			0
72	Korean: 26130666	D842V	0.2	1			1
73	Korean: 26130666	D842V	2.6	1			1
74	Korean: 26130666	D842V	3.8	1			1
75	Korean: 26130666	D842V	0.4	0			0
76	Korean: 26130666	D842V	3.9	1			1
77	Korean: 26130666	D842V	0.8	1			1
78	Korean: 26130666	I843del	10.1		0		
79	Korean: 26130666	D842del	25.2			1	1
80	Korean: 26130666	I843_D846del	3.9		0		

81	Korean: 26130666	I843_D846del	29.5		1		
82	European Sarcoma Centers	D842V	0.72	1			1
83	European Sarcoma Centers	D842V	2.6	1			1
84	European Sarcoma Centers	D842V	2.01	1			1
85	European Sarcoma Centers	D842V	26.99	1			1
86	European Sarcoma Centers	D842V	5.85	1			1
87	European Sarcoma Centers	D842V	13.25	1			1
88	European Sarcoma Centers	D842V	1.91	1			1
89	European Sarcoma Centers	D842V	44.75	1			1
90	European Sarcoma Centers	D842_D846delinsE	35.05		1		
91	European Sarcoma Centers	I843_D846del	28.04		1		
92	European Sarcoma Centers	D842_H845del	48.03		1		
93	European Sarcoma Centers	D846Y	46.75		1		
94	European Sarcoma Centers	D842_M844del	28.08		1		
95	European Sarcoma Centers	I843_S847delinsM	9.5		1		
96	European Sarcoma Centers	I843_D846del	28.04		1		
97	European Sarcoma Centers	D842_H845del	63.06		1		
98	European Sarcoma Centers	I843_S847delinsT	13		1		
99	OHSU	D842_D846delinsE	16		1		
100	OHSU	D842_I843delinsV	2			1	1
101	OHSU	D842_H845del	8		0		
102	MD Anderson	D842V	2.57	1			1
103	MD Anderson	D842V	26.60	1			1
104	MD Anderson	D842V	9.30	1			1
105	MD Anderson	D842V	2.13	1			1
106	MD Anderson	D842V	5.20	1			1
107	MD Anderson	D842V	12.37	0			0
108	MD Anderson	D842V	1.60	1			1
109	MD Anderson	D842V	3.80	1			1
110	Memorial Sloan Kettering	D842V	2	1			1
111	Memorial Sloan Kettering	I843_D846del + V658G*	29		1		
112	B2222 Study: 14645423	D842V	9.6	1			1
113	B2222 Study: 14645423	D842V	2.93	1			1
114	B2222 Study: 14645423	D842V	2.63	1			1
115	B2222 Study: 14645423	D842_H845del	89.49		1		
116	B2222 Study: 14645423	I843del	2.73		1		

Supplementary Table 7: Clinical responses of advanced PDGFRA-mutant GIST patients treated with first-line imatinib. Table includes corresponding PubMed ID of published data (if applicable), mutation information, progression free survival (PFS) in months, and corresponding censoring information. Treatment stop due to progression or death is designated as 1, ongoing treatment or treatment stop due to other factors (e.g. surgery) is designated as 0. *indicates a secondary mutation that was seen after imatinib progression.
